# A lightweight multi round confusion-diffusion cryptosystem for securing images using a modified 5D chaotic system

**DOI:** 10.1038/s41598-025-13290-y

**Published:** 2025-08-30

**Authors:** T. Anujaa, Afra Fathima Thajudeen Ali Ahamed, Vedika Baranwal, V. Thanikaiselvan, S. Subashanthini, C. Sivaranjani Devi, Amirtharajan Rengarajan

**Affiliations:** 1 School of Electronics Engineering, Vellore Institute of Technology (VIT), Vellore, 632 014 India; 2 School of Computer Science Engineering and Information Systems, Vellore Institute of Technology (VIT), Vellore, 632 014 India; 3 School of Electrical and Electronics Engineering, SASTRA Deemed University, Thanjavur, 613401 India

**Keywords:** Image encryption, Chaotic maps, Confusion-diffusion, SHA-512, Digital security, Multimedia protection, Electrical and electronic engineering, Computer science, Information technology

## Abstract

In recent years, technological advancements have made the transmission of confidential information spooky. This research proposes a modified 5D chaotic map and a new image encryption algorithm based on an integrated chaotic system developed with SHA-512 hashing and a confusion-diffusion architecture. The modified 5D chaotic map provides randomness, and its performance is evaluated through a bifurcation diagram and Lyapunov exponent. The randomness of chaotic sequences is validated through the NIST test. The multi-round diffusion and permutation incorporating the proposed chaotic sequences significantly enhances security by destroying pixel correlation among pixels. The encryption algorithm is validated through performance metric analysis, yielding NPCR of 99.6069%, UACI of 33.4284%, and entropy of 7.99442. These values depict advanced security features needed for various multimedia, medical, and military applications. Therefore, this approach reveals the extent to which chaotic encryption systems provide digital image protection in high-risk communication environments.

## Introduction

In the era of unlimited digital connections and the big data explosion, images have become the primary format for exchanging information. Images are crucial to social media and online communication systems, as well as satellite imaging, telemedicine, biometric systems, and surveillance networks. The urgency and importance of image security have never been greater, given that sensitive content is routinely transferred over potentially compromised digital channels. According to a data breach report released by IBM^1^ (2024), the average data breach cost for the respective year is USD 4.88 million, representing the biggest jump since the pandemic. Thus, there is an increasing demand for protecting multimedia information, which has triggered significant research into developing efficient, scalable, and secure methods for image encryption. Although the study focuses on providing security for confidential information in images, this raises the need for developing an efficient cryptographic system to ensure the secure transmission and storage of images^2^. The goal is to create a highly secure cryptosystem with advanced knowledge in image encryption and offer insights that could pave the way for introducing new methodologies for trending applications, ensuring safe and efficient image processing in an interconnected and data-driven world.

The growing concern about new threats to online communication platforms has led to a renewed emphasis on developing advanced image encryption algorithms. Traditional encryption techniques, such as the Advanced Encryption Standard (AES), Rivest-Shamir-Adleman (RSA), and Data Encryption Standard (DES), lack both efficiency and security. Hence, developing a new one with advanced security is necessary. To settle this issue, the recent trend is that encryption algorithms are based on chaotic systems^2^. Advanced modern image encryption techniques involve a set of functions that combine permutation and substitution operations with diffusion and confusion to achieve a secure cryptosystem.

Encryption techniques based on chaos^2,3^ have attracted much attention due to the characteristics of chaotic systems, such as sensitivity to initial conditions, pseudo-randomness and ergodicity. Due to these features, chaotic systems can be utilised in the confusion and diffusion processes, which are important for secure encryption. In recent years, many one-dimensional and multidimensional chaotic maps, including the Logistic Map, Henon Map, Arnold Cat Map, and Lorenz System^4,5^ have been thoroughly investigated and utilised in image encryption scheme design. The usual method employs a permutation based on value substitution, thereby breaking up the spatial correlation and uniformly redistributing pixel intensity. Recent studies on chaos-based techniques have yielded high key sensitivity and low correlation with cypher images, reinforcing the cryptographic robustness of the encryption procedure.

Simiao et al.^6^ propose a 4D (four-dimensional)chaotic system for the encryption algorithm, where chaotic sequences are utilised for DNA encoding. Also, the block scrambling methodology is employed to generate secure communication. Deep et al.^7^ discovered a novel 3D image encryption algorithm based on SHA-256, where the initial conditions are obtained from the SHA algorithm for the LDCML model, and this 3D model provides faster encryption. XueFeng et al.^8^ proposed a 4D hyper-chaotic system developed from a 3D Lorenz chaotic system, and the validation of the equation is performed using STM32. Then, the chaotic system is applied to image encryption. Chaos-based image encryption was designed by Talha et al.^7^ to secure multimedia content in cloud storage. A modified skew tent map is employed. It involves two rounds of permutation and diffusion for cloud storage systems. Moatsum et al.^9^ describe their newly created perturbed logistic chaotic map and permutation operation, demonstrating high security and low computational time performance.

The existing paper primarily develops low-dimensional chaotic maps designed for lightweight image encryption schemes. There is a lack of inspection of the complex behaviour of higher-dimensional chaotic maps. Some chaos-based encryption provides low key sensitivity and a small key space. The insufficient integration of image encryption with next-generation technologies, such as blockchain, quantum computing, 5G, and AI algorithms, has yet to be thoroughly examined. While realising real-time applications, acute observation of resource constraints, speed, time complexity, and the complexity of pseudo-random number generation has been limited. While encryption and compression are each essential for effective image transfer, neither the trade-offs between compression compatibility nor cryptographic integrity are explored. Securing key distribution through a complex and chaotic system remains an area of ongoing research for resource-constrained environments. Although existing papers demonstrate better performance in many aspects, there is a lack of lightweight models and higher-dimensional chaotic equations. Our research addresses security risks in such systems and evaluates methods for enhancing image protection in a highly vulnerable communication environment. This work proposes an advanced image encryption technique featuring a multi-round confusion-diffusion structure and enhanced security features.

The Contribution of the research is listed below.

i. A modified 5D chaotic map equation derived from the Lorenz equation has been introduced, which bestows outstanding complex and dynamic behaviour, providing chaotic responses that validate the randomness of the sequences.
ii. Implemented a multi-round permutation and diffusion framework using the proposed modified 5D chaotic map equation, which provides strong security through an encryption algorithm.
iii. Additional security for the encryption algorithm is achieved through the secondary image at the last stage of the architecture.
iv. A simulation performance analysis has been conducted, demonstrating significant improvements in resistance to statistical, differential, and brute-force attacks.

The paper structure is as follows: Section “Proposed methodology for the image encryption cryptosystem” elaborates on the modified 5D chaotic map equation and the encryption algorithm using a confusion-diffusion architecture. In Section “Performance assessment of the 5D chaotic map equation”, the performance analysis of the chaotic map equation is demonstrated. The results and the simulation analysis of the encryption algorithm are validated in Section “Experimental evaluation of the proposed encryption algorithm”. The conclusion and future work are outlined in Section “Conclusion and future work”.

## Proposed methodology for the image encryption cryptosystem

The proposed algorithm defines the cryptosystem using two sections: the chaotic sequence generation using the modified 5D chaotic map equation and the image encryption algorithm. This is elaborately given in Sections “Modified 5D chaotic equation” and “Initial seed generation process”

### Modified 5D chaotic equation

The newly created five-state coupled differential equations, the modified 5D chaotic map equation, are listed in the Eqs. (1–5). The defining control parameters like ‘m, l, T, q, e, t, and a’ constitute these equations to govern the evolution of the state variables d1, d2, d3, d4, and d5.

1 $$d_{1}{\prime} = l\left( {d_{2} - d_{1} } \right) + d_{4} + esin\left( {d_{5} } \right)$$

2 $$d_{2}{\prime} = qd_{1} - d_{2} - d_{1} d_{3} + 0.1cos\left( {d_{5} } \right)$$

3 $$d_{3}{\prime} = - Td1 + d_{1} d_{2} + 0.1sin\left( {d_{4} } \right)$$

4 $$d_{4}{\prime} = td_{4} - d_{1} d_{3} + 0.1cos\left( {d_{3} } \right)$$

5 $$d_{5} ^{\prime} = - ad_{5} + d_{1} + 0.1sin\left( {d_{2} } \right)$$

where m, l, T, q, e, t are the control parameters and d_1,_ d_2,_ d_3,_ d_4,_ d_5_ are the state variables. The modified 5D chaotic map equation uses five coupled differential equations ${{d}_{1}}{\prime}, {{d}_{2}}{\prime}, {{d}_{3}}{\prime}, {{d}_{4}}{\prime} {, {d}_{5}}{\prime}$. Equation 1 is obtained by the linear damping or feedback term resulting from the coupling between d1 and d2 with the sinusoidal term, which introduces complexity to the sequence. Equation 2 includes a nonlinear interaction and linear damping between d1 and d3, with a cosine term to provide good randomness in the chaotic system. Equation 3 defines that $-Td1$ is the control term with the nonlinear growth term d1 and d2 with the sine function. Equation 4 says that $t{d}_{4}$ is the decay function controlling the control parameter t and has a cross-coupling term ${d}_{1}{d}_{3}$ which enhances complexity through the use of the cosine function. $-a{d}_{5}$ denotes the decay term with the linear influence from the term ${d}_{1}$ along with the trigonometric function sine term in Eq. 6. Thus, Eqs. (1–5) are collectively used in a nonlinear system with feedback loops and also with strongly coupled terms. The inclusion of trigonometric functions, such as sine and cosine terms, ensures a strong chaotic behaviour and sensitive initial conditions. The initial and control parameters are obtained by utilising the input image to guarantee secure communication.

### Initial seed generation process

*Step 1* Obtaining initial parameter and control parameter values.

The first step of the framework involves calculating the initial and control parameters. The control parameters of the proposed 5D chaotic map are m, l, T, q, e, t, and a. The desired system parameters value generation and the initial state vector are obtained from the hash of the input image.

*Step 2* Hashing.

The hashing process is performed using SHA-512, and the image is used as input for the hashing operation. SHA-512 is a hash function that takes the input as an image and is essentially integrated into the encryption process for generating five initial parameters and seven control parameters of the modified 5D chaotic map equation. SHA-512 provides irreversible and sensitive initial conditions, where different hashes are produced for different inputs. So the key generated are not easy to guess. The SHA-512 generates a 64-byte hash (denoted as h) using a 512-bit secret key. This hash is used to extract the control parameter values and initial states by doing manipulations within the chaotic system. The first 40 bytes of the hash are allocated to extract the control parameter values, and the remaining 20 bytes are used to extract the initial state values. Eqs (6–12) involves manipulating steps to get the desired parameter values.

6 $$m = 0.1 + \frac{{xor\left( {h1:h4} \right)}}{2048}$$

7 $$l = 0.5 + \frac{{xor\left( {h5:h8} \right)}}{1024}$$

8 $$T = 0.2 + \frac{{xor\left( {h9:h12} \right)}}{2048}$$

9 $$q = 0.3 + \frac{{xor\left( {h13:h16} \right)}}{1024}$$

10 $$e = 0.15 + \frac{{xor\left( {h17:h20} \right)}}{2048}$$

11 $$t = 0.25 + \frac{{xor\left( {h21:h24} \right)}}{1024}$$

12 $$a = 0.4 + \frac{{xor\left( {h25:h28} \right)}}{1024}$$

Thus, the obtained values are bounded within some given ranges. Table 1 specifies the parameter ranges for the above chaotic map equation, which limits the complexity and unpredictability of the generated chaotic sequence. Obtaining the correct parameters and initial states from the image data ensures that encryption is directly related to the input-specified version, thereby instigating further security.

**Table 1:** Parameter ranges.

Name	Value
M	0.1–0.3
L	0.5–0.8
T	0.2–0.4
Q	0.3–0.6
E	0.15–0.35
T	0.25–0.5
A	–0.7

### Chaotic sequence generation

The next step is the generation of a chaotic sequence matrix to be used for both the encryption and decryption processes. It first initialises the chaotic system’s states and iteratively computes new state values with the Multi-Wing Butterfly chaotic map. Subsequently, the chaotic sequence is normalised and transformed to ensure its chaotic nature and not diverge in the long run. Hence, the formed chaotic sequence matrix will consist of values for driving diffusion and permutation operations while encryption and decryption are being carried out. the chaotic sequence generated using Eq. 1is d1, d2, d3, d4 and d5.

### Implementation of the confusion diffusion framework

**Fig. 1:**
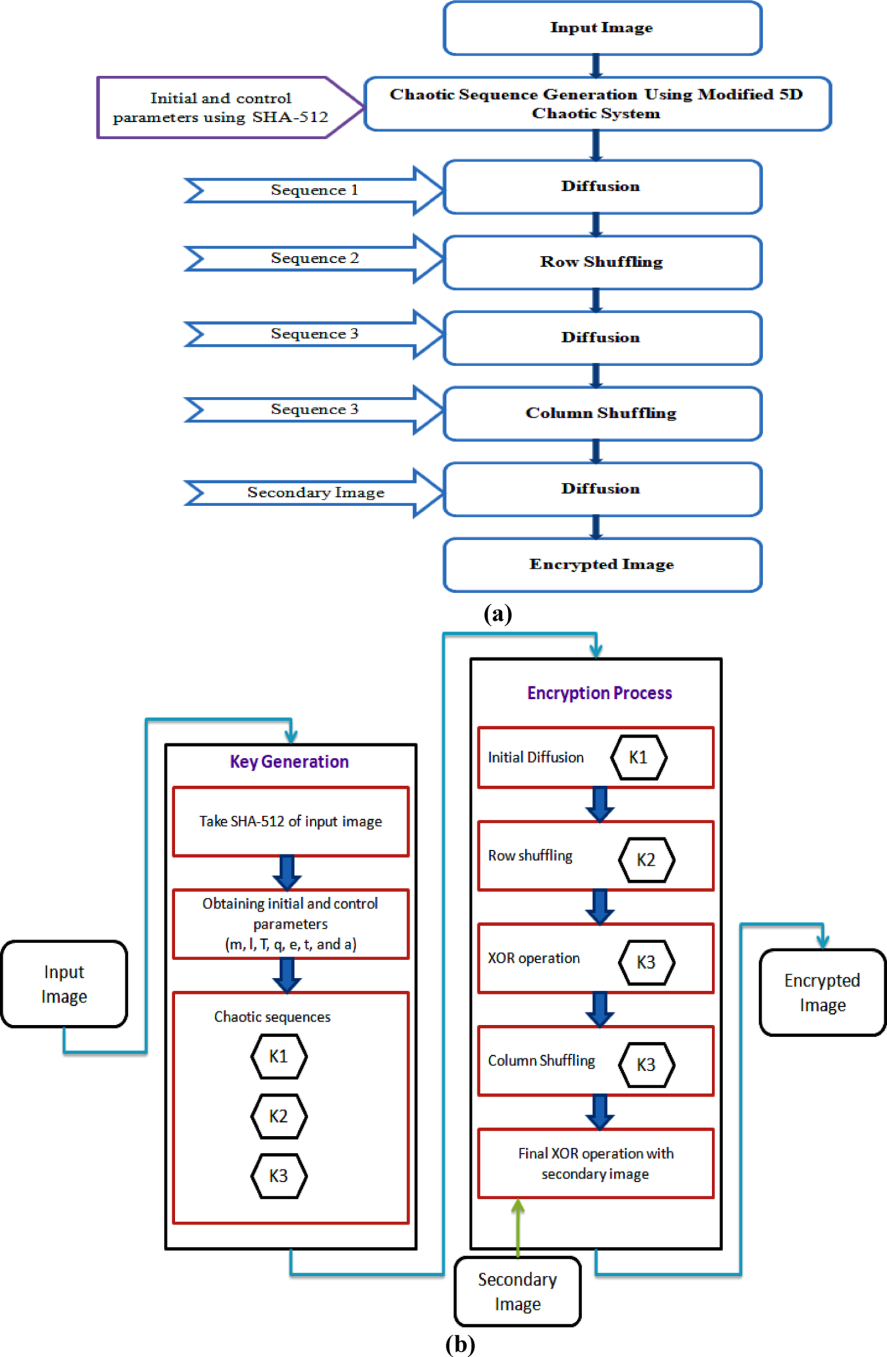

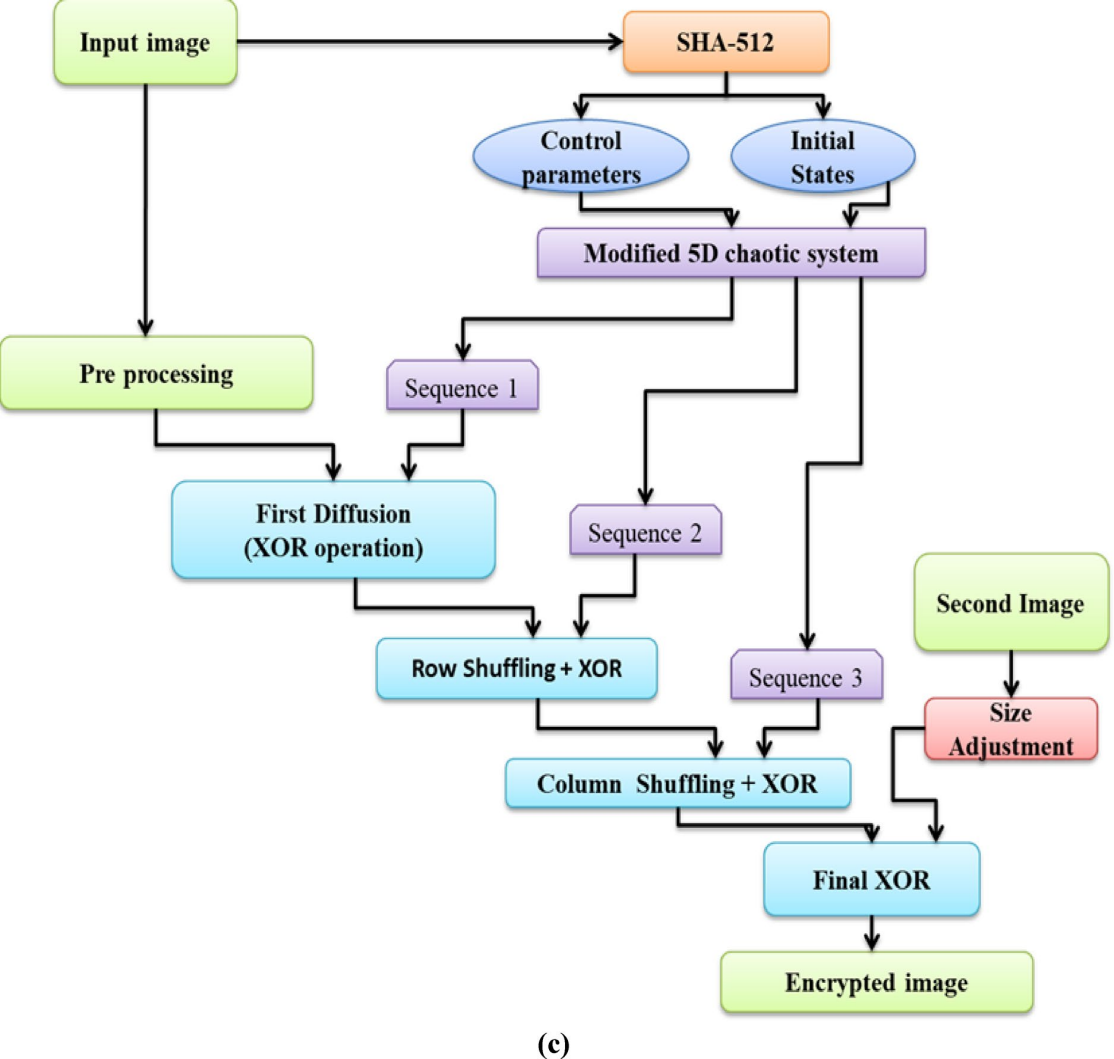
(**a**) Steps for proposed system confusion diffusion mechanism. (**b**) Flow diagram of the proposed methodology. (**c**) Encryption algorithm architecture.

The core methodology is the image encryption process, which consists of multiple rounds of diffusion and permutation, utilising a chaotic sequence accordingly. The initial step will be to reshape the chaotic sequence to compare it with the dimensions of the input image. The encryption algorithmic process of the proposed algorithm is given in the following steps (a–g). The precise structure of the confusion diffusion mechanism is discussed in Fig. 1a, and the flow diagram of the proposed methodology, where the input image is transformed into an encrypted image, is given in Fig. 1b.

The detailed image encryption algorithmic steps, which involve confusion and diffusion processes, are listed below. Figure 1c shows the proposed encryption algorithm architecture.

*Step 1* Image encryption using multi-rounds of confusion and diffusion.

- Initialisation.
  The encryption algorithm inputs are taken as IM, CS, SI
  IM: Input image
  CS: Chaotic sequence
  SI: Secondary image

b. Chaotic sequence generation.

The required chaotic sequence is obtained using the control and initial parameter formulation in Section “Chaotic sequence generation”. The chaotic sequence thus generated is iterated 10,000 times and normalised for further confusion diffusion steps.

c. Initial diffusion.

The initial diffusion of pixels of the input image is strong enough to provide security at the initial stage of communication. Therefore, the input image pixels is now xor-ed with chaotic sequence 1 (CS1) of the modified 5D chaotic map equation.

13 $$D1 = xor\left( {IM, CS1} \right)$$

where IM is the original image, and CS1 is the chaotic sequence

d. Row shuffling.

Row shuffling is a process where image pixels of the rows are rearranged based on chaotic sequence. For row-wise shuffling, the chaotic sequence 2 of the proposed equation is used to shuffle the rows of D1.

14 $${\text{D2 }} = {\text{ Rowshuffle }}\left( {{\text{D1}},{\text{ CS2}}} \right)$$

where D1 is the result of initial diffusion, and CS2 is the chaotic sequence

e. Xor-ing row shuffled matrix.

For the concept of strong diffusion, again,the resultant confused matrix D2 undergoes a diffusion process with the same chaotic sequence CS2

15 $$D3 = xor\left( {D2, CS2} \right)$$

where D2 is the row shuffled pixel matrix

f. Column Shuffling.

Another shuffling is performed based on the column to achieve stronger security. This step involves input as D3 and chaotic sequence CS3

16 $$D4 = Columnshuffle\left( {D3,CS3} \right)$$

where D3 is the previous step output

g. Xor-ing column shuffled matrix.

Now the column shuffled matrix D4 is xor-ed with chaotic sequence CS3

17 $$D5 = D4 xor CS3$$

where D4 is the column shuffled matrix

*Step 2* Adding additional complexity.

Further complexity is added to the resultant matrix of step 1(g) to ensure a strong encryption algorithm. Here, a new step called a secondary image (SI) is introduced in the final image to create a stronger diffused image. Before the addition of a secondary image, size adjustment for the secondary image is performed

18 $$D6 = D5 xor SI$$

where D5 is the resultant matrix of step 1(g) and SI is the secondary image.

**Fig. 2:**
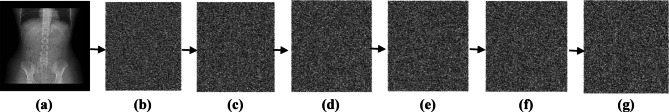
(**a**) Original image, (**b**)initial diffusion using S1, (**c**) row shuffling using S2, (**d**) xor-ing row shuffled matrix with S2, (**e**) column shuffling using S3, (**f**) xor-ing column shuffled matrix with S3, (**g**) final encrypted image using a secondary image.

The resultant encrypted image is obtained by the end of the above steps. The step-by-step outcome of the proposed encryption algorithm is shown in Fig. 2**.**

## Performance assessment of the 5D chaotic map equation

To examine the proposed chaotic map equation, this section demonstrates the chaotic performance behaviour of the modified 5D chaotic map equation. The performance metrics^10^, such as the phase diagram and the NIST test, are evaluated to analyse their randomness, sensitivity, chaotic nature, and complexity.

### Phase diagram

**Fig. 3:**
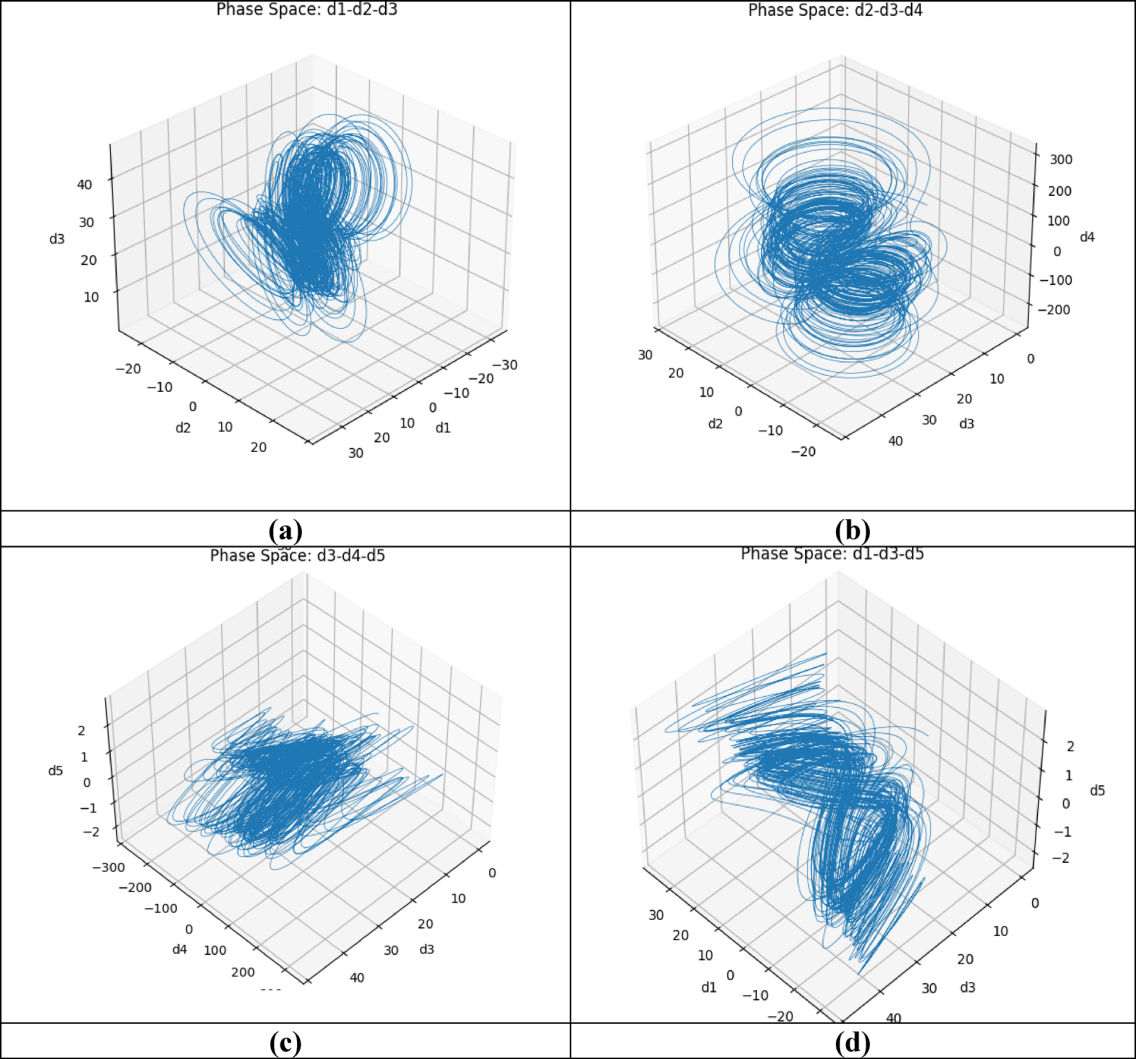
Phase diagram of the chaotic system. (**a**) phase diagram of the d1-d2-d3 plane, (**b**) phase diagram of the d2-d3-d4 plane, (**c**) phase diagram of the d3-d4-d5 plane, (**d**) phase diagram of the d1-d3-d5 plane.

The phase diagram is the visual system to see the system entering chaos where it is sensitive to its initial conditions. It is a graph plotted between the parameters. Hence, the plot is between d1, d2, d3, d4 and d5. Figure 3a shows the 3D phase diagram of the modified chaotic map equation, where Fig. 3a shows the phase portrait of d1, d2 and d3 sequences. Similarly, Fig. 3b shows the phase portrait of d2, d3 and d4, Fig. 3c shows the phase portrait of d3, d4 and d5, and Fig. 3d shows the phase portrait of d1, d3 and d5.

### NIST test

National Institute of Standards and Technology (NIST)^10,11^ SP800-22checks the randomness of the chaotic sequence generated by the proposed modified 5D chaotic map equation. The NIST test consists of 15 randomness tests. The test can be evaluated by using 1000000 bits. Every test in NIST is passed only if the probability of each test, represented as a P value, is larger than 0.01. Table 2 presents the NIST test results for the modified 5D chaotic sequence, which demonstrate its randomness and suitability for security applications. Table 2 shows that the P values of all the tests are greater than 0.01, confirming the randomness of the chaotic sequences.

**Table 2:** NIST test results.

No	Type of test		P value	Conclusion
1	Frequency test (monobit)		0.6326502050386464	Random
2	Frequency test within a block		0.3132675930111212	Random
3	Run test		0.26281103127028804	Random
4	Longest run of ones in a block		0.6100305089456537	Random
5	Binary matrix rank test		0.7728446477849531	Random
6	Discrete fourier transform (spectral) test		0.659590791427404	Random
7	Non-overlapping template matching test		0.5579675671836456	Random
8	Overlapping template matching test		0.058492039130392075	Random
9	Maurer’s universal statistical test		0.9578043118723146	Random
10	Linear complexity test		0.06822107775129024	Random
11	Serial test		0.09063711502208262	Random
			0.2279965495409031	Random
12	Approximate entropy test		0.16342628009051988	Random
13	Cummulative sums (forward) test		0.47598695982883577	Random
14	Cummulative sums (reverse) test		0.806686397335922	Random
15	Random excursions test:			
	State	Chi squared	P value	Conclusion
	− 4	2.708034400504492	0.7448893399207672	Random
	− 3	2.289710344827586	0.031026641907626804	Random
	− 2	10.467703858508456	0.06301715716571302	Random
	− 1	8.949843260188088	0.11108154316939484	Random
	+ 1	4.015673981191222	0.5471614935196246	Random
	+ 2	9.185301288749564	0.1018974868657128	Random
	+ 3	12.041434482758623	0.03422442210762175	Random
	+ 4	14.640335335720877	0.012014923404003137	Random
16	Random excursions variant test:			
	State	Counts	P value	Conclusion
	− 9.0	362	0.6796877019021097	Random
	− 8.0	334	0.878135773535464	Random
	− 7.0	322	0.973721488445495	Random
	− 6.0	298	0.8020646815134346	Random
	− 5.0	286	0.6632037695651256	Random
	− 4.0	280	0.5594985352899394	Random
	− 3.0	294	0.6580305640790922	Random
	− 2.0	302	0.6975885118672047	Random
	− 1.0	301	0.47607670840662175	Random
	+ 1.0	363	0.08151252467043998	Random
	+ 2.0	456	0.0017392781098894256	Non-random
	+ 3.0	511	0.000675268789706703	Non-random
	+ 4.0	542	0.0008471300999453507	Non-random
	+ 5.0	552	0.0021060648501195775	non-random
	+ 6.0	473	0.06601967502129268	Random
	+ 7.0	384	0.4753968053711022	Random
	+ 8.0	349	0.7590977958297784	Random
	+ 9.0	303	0.877898928289632	Random

According to Table 2, the frequency test checks the number of zeros and ones to ensure a balanced chaotic sequence. Similarly, the other tests checks the chaotic sequences periodic patterns, checks for clustering of bits, verifies the length of the longest sequence, linear dependence between sequences, checks for specific predefined patterns, no repeating sub patterns, verifying the complexity, frequency of all overlapping and non overlapping bits, checking repeated patterns, monitors in random walk and analysing the number of visits to various tests during random walk. The experiment outcomes validate that the results pass the P value, which is greater than or equal to 0.01. Thus, the chaotic sequences generated using the modified 5D chaotic system are highly suitable for cryptographic applications.

### Bifurcation diagram and Lyapunov exponent

**Fig. 4:**
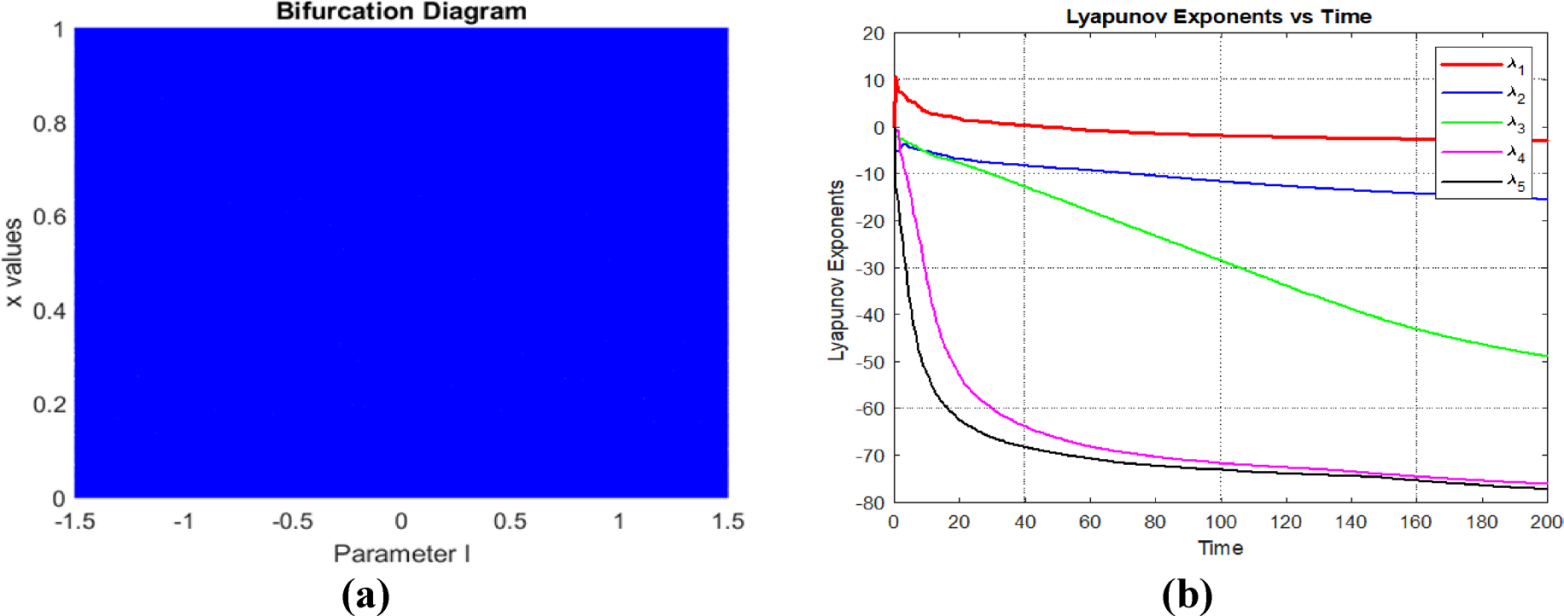
(**a**) Bifurcation diagram and (**b**) Lyapunov exponent.

Bifurcation diagrams^5^ and Lyapunov exponents^12^ are important aspects to analyse the randomness of chaotic sequences. A bifurcation diagram provides a graphical representation of a dynamic system’s chaotic behaviour. The proposed modified 5D chaotic system enter chaotic behaviour in the entire region. The Lyapunov exponent demonstrates the system’s behaviour as it enters a chaotic state. If the system yields a positive value, then the system is considered a chaotic system. On the other hand, if two or more positive values occur, it denotes hyperchaotic behaviour. Fig 4 shows the Bifurcation diagram and Lyapunov exponent of the proposed modified 5D chaotic system.

## Experimental evaluation of the proposed encryption algorithm

The proposed multi-round confusion and diffusion architecture, utilising a modified 5D chaotic map equation, is simulated using MATLAB R2024a on a PC with an Intel(R) Core (TM) i3-1005G1 CPU and 4 GB of RAM, running Microsoft Windows 11. One hundred samples, comprising DICOM images, satellite images, fingerprint images, and QR images, were used to conduct this analysis. The sample images were downloaded from the following sources: https://sipi.usc.edu/database/database.php?volume=aerials, https://www.kaggle.com/datasets/samahsadiq/benign-and-malicious-qr-codes, https://www.dicomlibrary.com/, http://bias.csr.unibo.it/fvc2000/download.asp. Then, the analysis was conducted for statistical attacks, differential attacks, brute force attacks, and encryption quality to assess the quality of the encryption algorithm.

### Statistical analysis^13^

The proposed encryption algorithm is validated through statistical analysis, including histograms, correlation analysis, entropy, and the chi-square test.

#### Histogram analysis

**Fig. 5:**
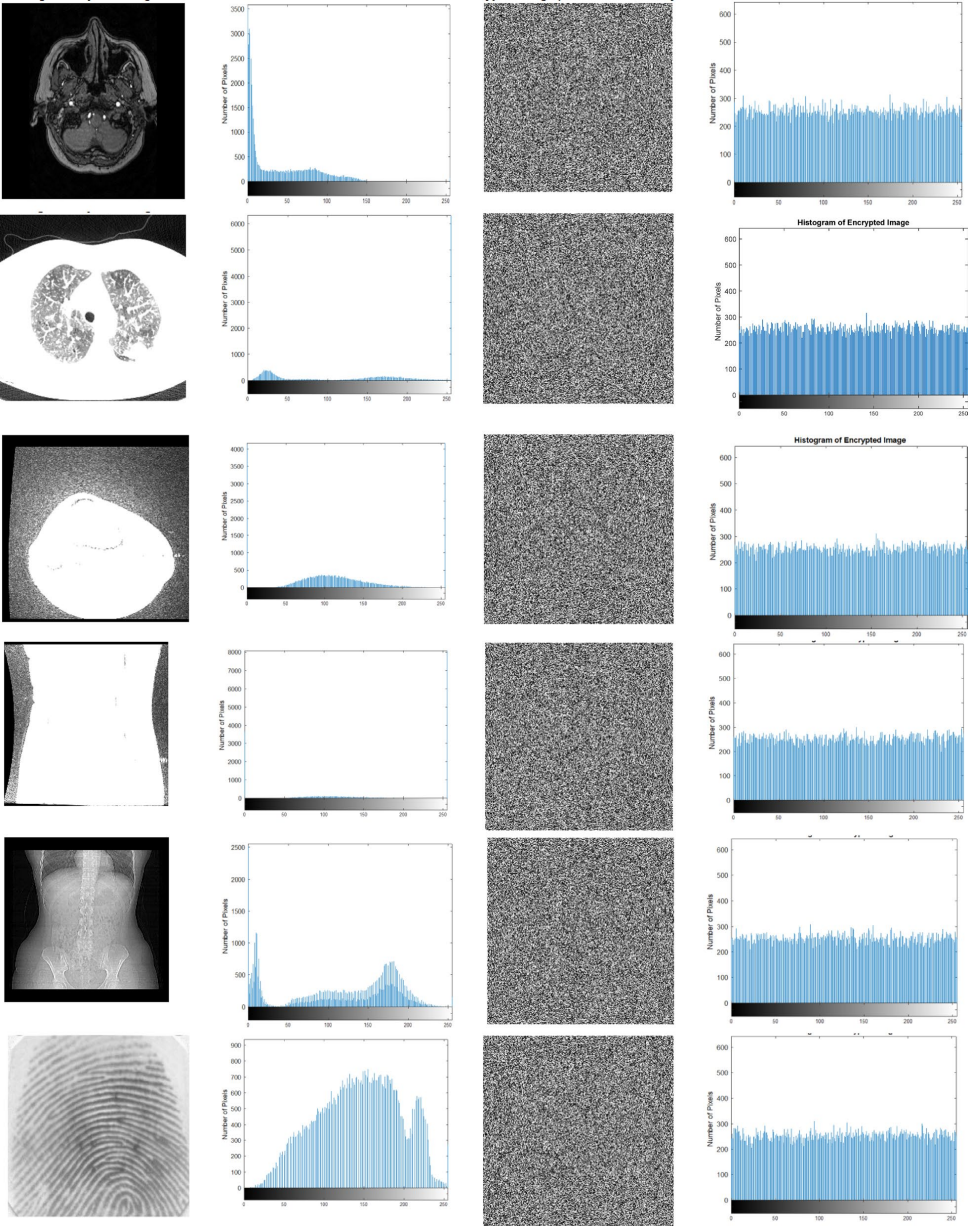

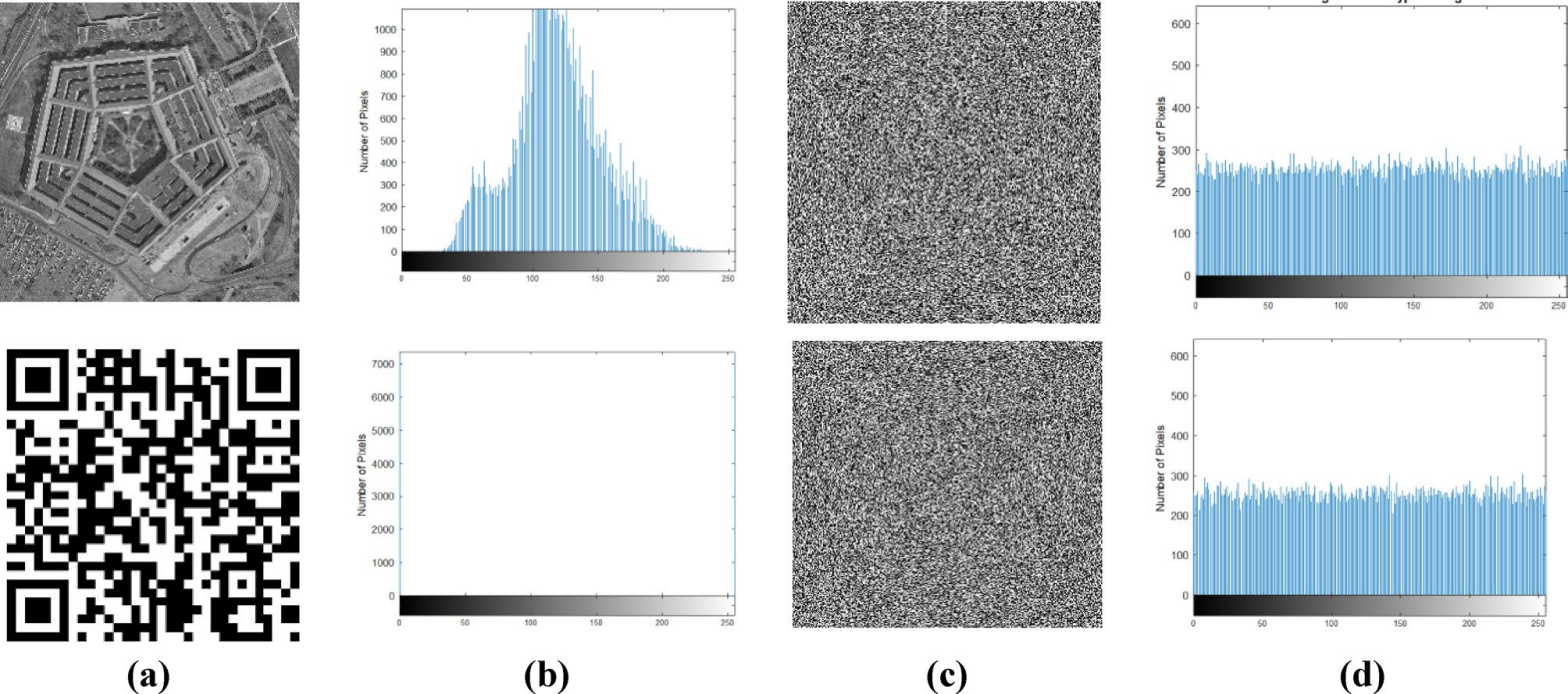
(**a**) Original image, (**b**) Histogram of original image, (**c**) Encrypted image, (**d**) Histogram of encrypted image.

Histogram^14^ is the graphical representation of the image, where the pixel distribution of the image provides a pictorial clue that reveals susceptibility to statistical attacks. Hence, the encrypted image has a uniform pixel distribution, and the flat image provides resistance to statistical attack. Figure 5 shows the original image, its histogram, the encrypted image, and the histogram of the encrypted image. It is ensured that the encrypted images of the samples offer a uniform distribution of pixels. Thus, the cipher image does not leak any information due to the even distribution of pixels.

#### Correlation coefficient analysis

**Fig. 6:**
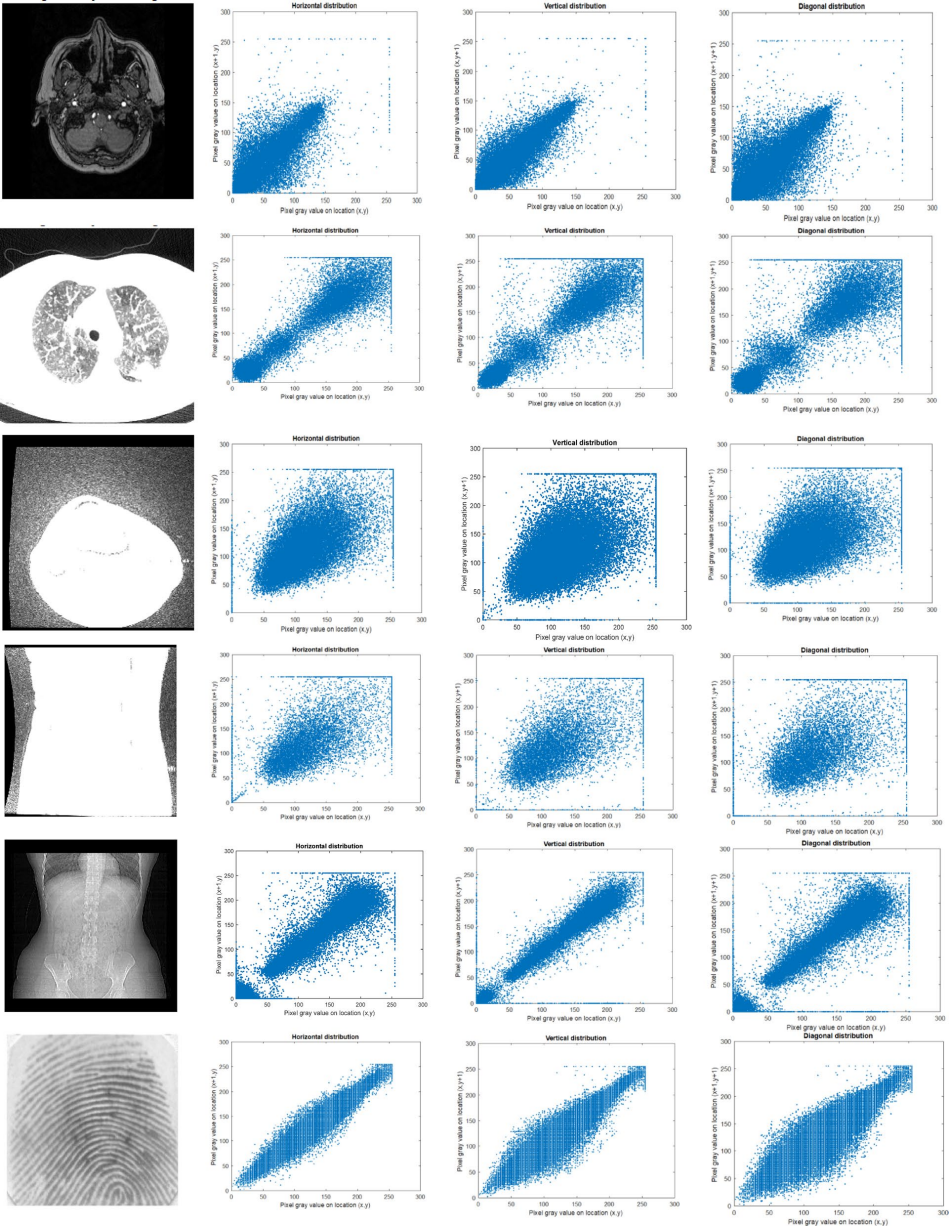

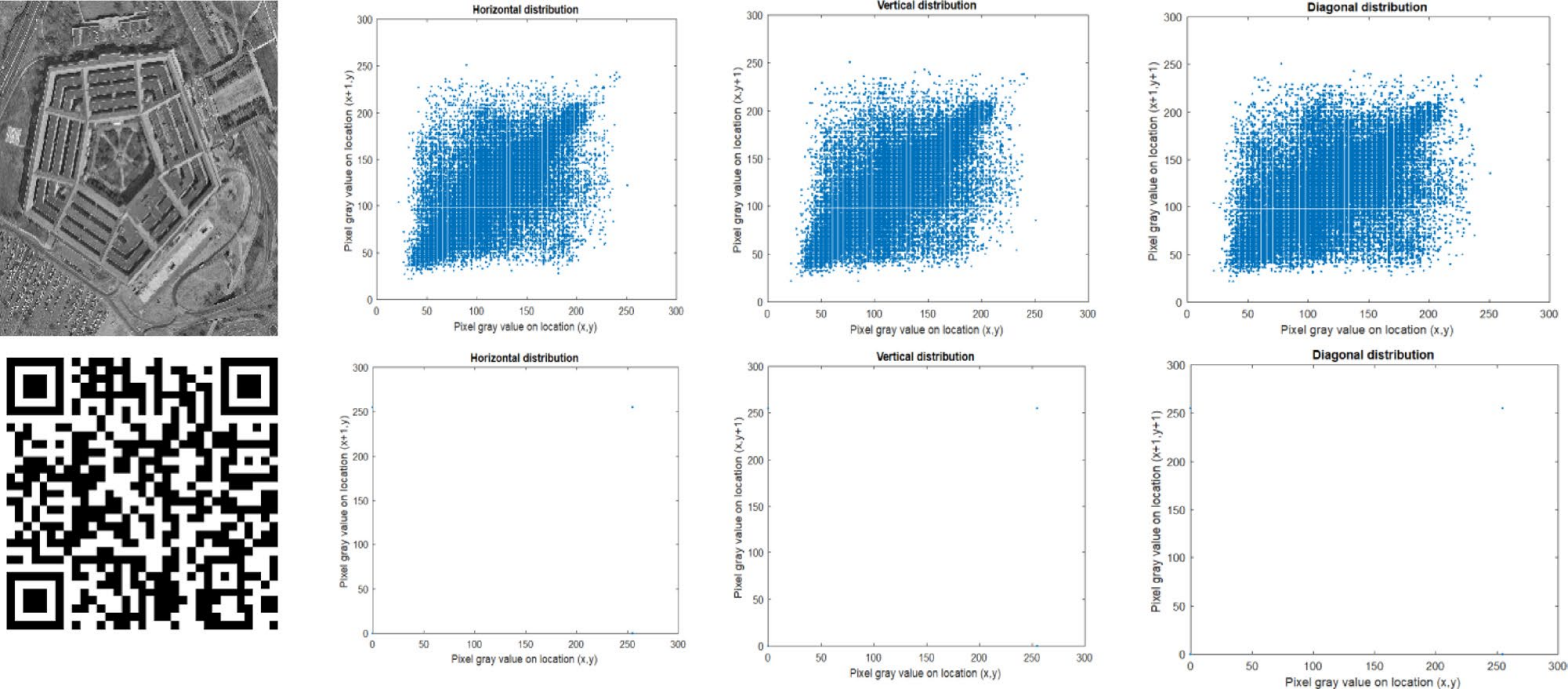
Original image correlation analysis (**a**) Original image (**b**) Horizontal, (**c**) Vertical, (**d**) Diagonal.

**Fig. 7:**
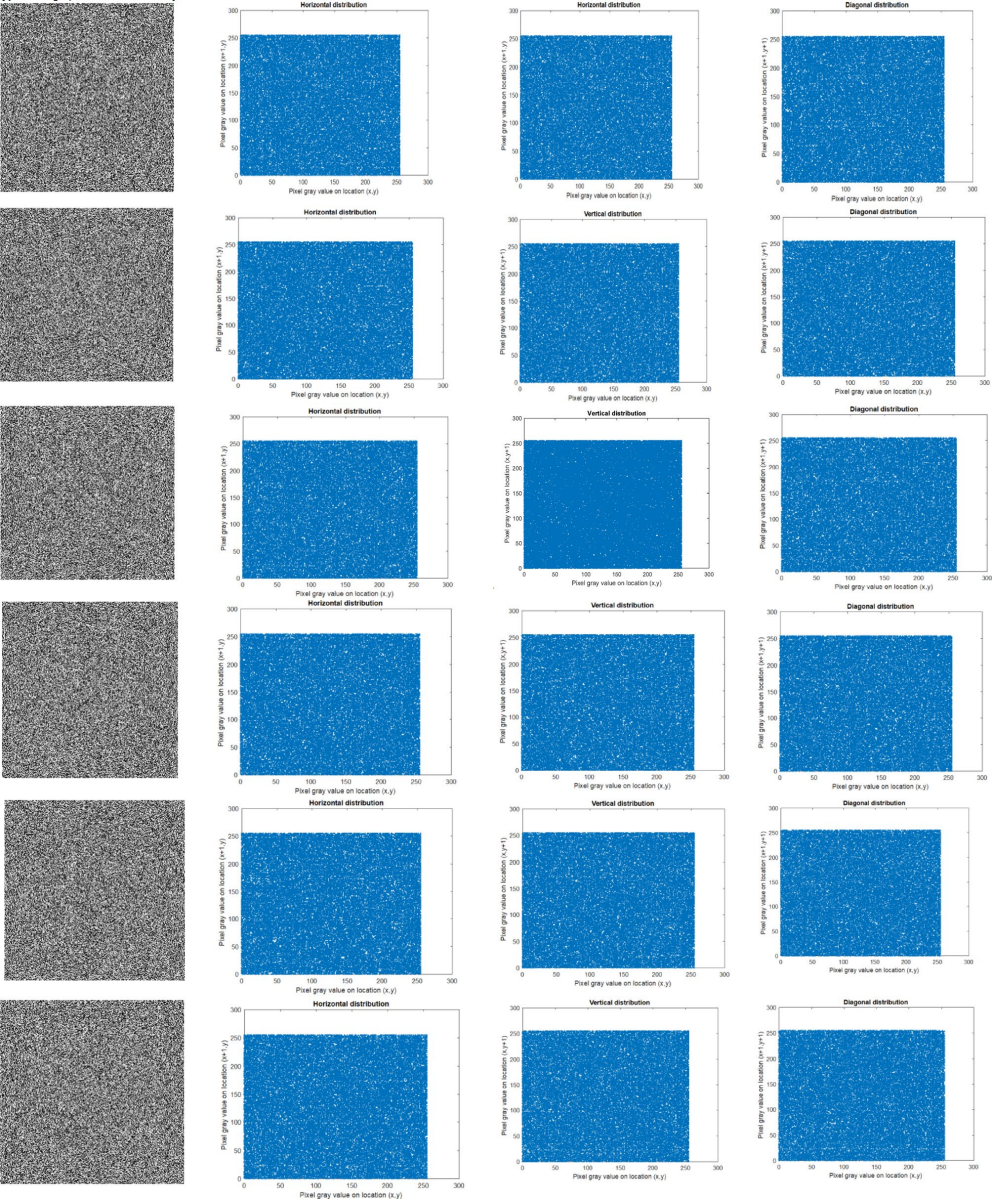

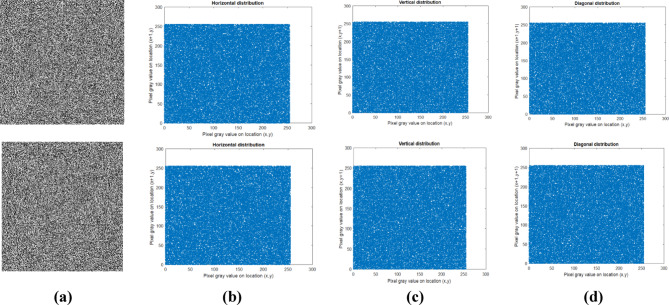
Encrypted image correlation analysis (**a**) Encrypted image (**b**) Horizontal, (**c**) Vertical, (**d**) Diagonal.

The correlation coefficient^11^ measures the relationship between adjacent pixels, including horizontal, vertical, and diagonal. The encryption algorithm demonstrates its effectiveness by withstanding statistical attacks, as evidenced by checking the correlation coefficient. The encrypted image has a lower correlation value, which is nearer to zero, indicating better encryption quality. The correlation analysis is evaluated using Eqs. (19–22).

19 $$E\left( x \right) = \frac{1}{N}\mathop \sum \limits_{j = 1}^{N} x_{j}$$

20 $$D\left( x \right) = \frac{1}{N}\mathop \sum \limits_{j = 1}^{N} (x_{j} - E\left( x \right))^{2}$$

21 $$cov\left( {x,y} \right) = \frac{1}{N}\mathop \sum \limits_{j = 1}^{N} \left( {x_{j} - E\left( x \right)} \right)\left( {y_{j} - E\left( x \right)} \right)$$

22 $$corr\left( {x,y} \right) = \frac{{cov\left( {x,y} \right)}}{{\sqrt {D\left( x \right)D\left( y \right)} }}$$

where x, y represents the neighbouring pixels, $E\left(x\right)$ represents the expected mean, D(x) is the variance, $cov\left(x,y\right)$ denotes the covariance and $corr\left(x,y\right)$ is the correlation between the pixels x and y. Correlation analysis was performed on different types of images. The correlation analysis of the original image is depicted in Fig. 6, and the correlation analysis of the encrypted image is visualised in Fig. 7**.** The study was performed in horizontal, vertical, and diagonal directions. The figures show that the encrypted image values are closer to zero, as mentioned in Table 3.

**Table 3:** Correlation analysis of original and encrypted image.

Sample images	Original image			Encrypted image		
	Horizontal	Vertical	Diagonal	Horizontal	Vertical	Diagonal
Sample1	0.9110	0.9419	0.8912	0.0045	0.0101	0.0104
Sample 2	0.9783	0.9677	0.9586	− 0.0027	0.0017	0.0178
Sample 3	0.9565	0.9430	0.9409	0.0024	0.0042	0.0116
Sample 4	0.9667	0.9470	0.9370	− 0.0110	0.0041	0.0085
Sample 5	0.9775	0.9791	0.9661	− 0.0070	0.0091	0.0150
Sample 6	0.9695	0.9453	0.9137	0.0011	0.0022	0.0048
Sample 7	0.6052	0.6302	0.5335	− 7.3561e−04	4.0267e−05	0.0044
Sample 8	0.8786	0.8848	0.7769	− 0.0042	0.0022	0.0090
Sample 9	0.9614	0.9514	0.9499	5.2678e-04	0.1036	0.0114
Sample 10	0.9659	0.9579	0.9551	− 0.0040	0.0117	0.0087

#### Entropy

**Fig. 8:**
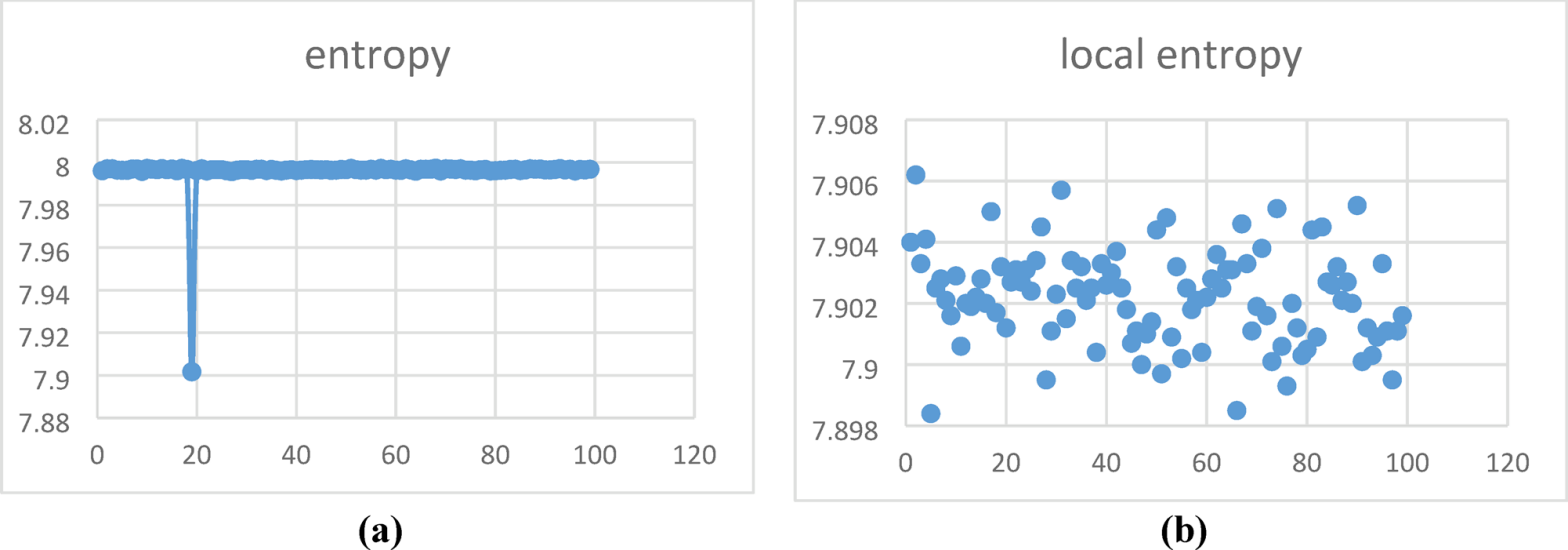
Entropy values of (**a**) Global, (**b**) Local.

The entropy^15^ value is evaluated to find the randomness and uncertainty of the pixels. The cipher image is said to be highly random only if it has a high entropy value 8. The entropy is calculated using the eq. (23).

23 $$H\left( k \right) = - \mathop \sum \limits_{i = 1}^{255} P\left( {k_{i} } \right)\log_{2} P\left( {k_{i} } \right)$$

where the probability of occurrences is represented by $P({k}_{i})$_._ Also, the local entropy is noted based on its critical values, where the block size is 32 with 1932 pixels per block. Thus, the actual randomness is calculated via the local entropy of the encrypted images, which are non-overlapping blocks. A sample of 100 image entropy values is evaluated, as shown in Fig. 8**,** and it is realised that the entropy value is closer to 8. Table 4 shows the global and local entropy values.

**Table 4:** Global and local Shannon’s entropy analysis.

DICOM	Encrypted image		Local entropy critical values		
	Global entropy	Local entropy with total number of blocks = 32	H^5%^left = 7.9019 H^5%^right = 7.9030	H^10%^left = 7.9017 H^10%^right = 7.9032	H^1%^left = 7.9015 H^1%^right = 7.9034
Sample 1	7.9961	7.9040	Pass	Pass	Pass
Sample 2	7.997	7.9062	Pass	Pass	Pass
Sample 3	7.997	7.9033	Pass	Pass	Pass
Sample 4	7.9964	7.9041	Pass	Pass	Pass
Sample 5	7.9963	7.8984	Pass	Pass	Pass
Sample 6	7.9963	7.9025	Pass	Pass	Pass
Sample 7	7.9969	7.9028	Pass	Pass	Pass
Sample 8	7.9969	7.9021	Pass	Pass	Pass
Sample 9	7.9958	7.9016	Pass	Pass	Pass
Sample 10	7.9972	7.9029	Pass	Pass	Pass

#### Chi-square test analysis

**Fig. 9:**
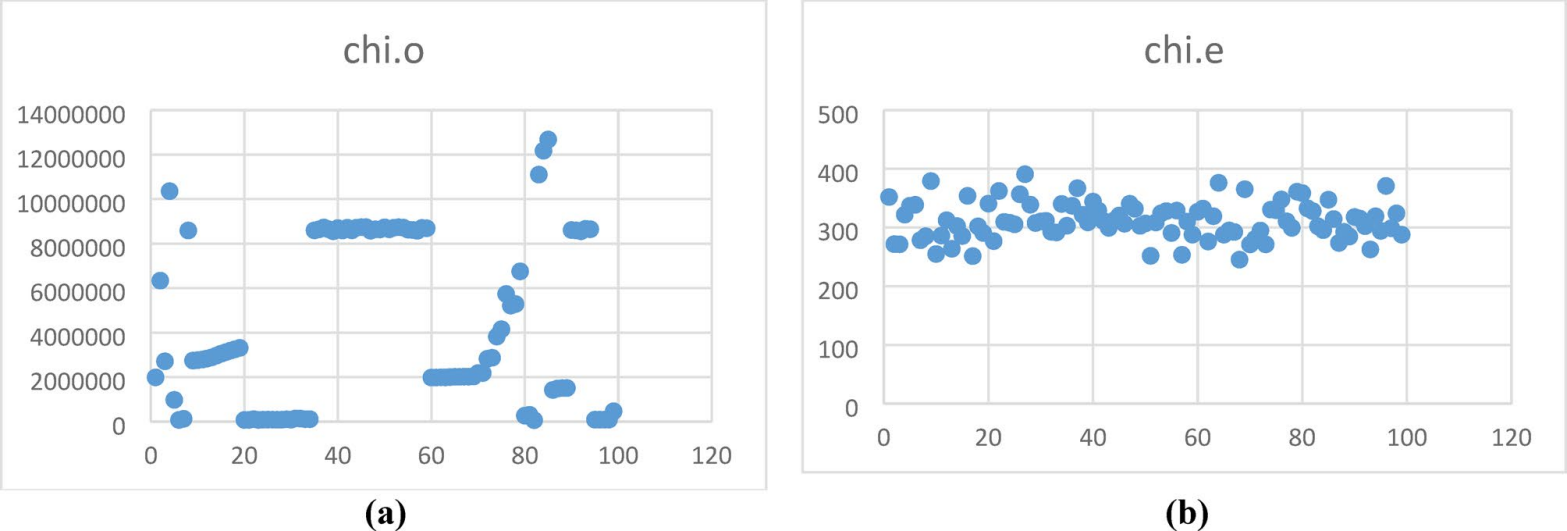
Chi square analysis of (**a**) Original image, (**b**) Encrypted image.

The pictorial representation of pixels of the original and the encrypted images are not yet satisfied. The chi-square test is evaluated to fill this gap and validate the encrypted image pixel uniformity. Chi-square^16^ is calculated using the Eq. (24).

24 $$\chi^{2} = \mathop \sum \limits_{i = 1}^{256} \frac{{\left( {O\left( i \right) - E\left( i \right)} \right)^{2} }}{E\left( i \right)}$$

where, O is the observed encrypted image histogram value. The expected value of the ideally encrypted image is denoted as E, and its value is 2^L^, where L is the image’s bit depth. The values are tested at the stated significance level and 255 degrees of freedom. According to null hypothesis theory, it is said that pixels are said to be uniformly distributed only if the chi-square value is less than the critical value. Table 5 presents the chi-square values for both the original and encrypted images. The chi-square values of encrypted images are lower than those of the original image. Figure 9 shows the analysis for 100 samples; its values are plotted as in the original and encrypted images.

**Table 5:** Chi square analysis of the original and encrypted image.

Dicom images	Chi-square for original image	Chi-square for encrypted image
Sample 1	1,988,614.7344	251.9922
Sample 2	6,336,085.8594	271.7109
Sample 3	2,711,718.4297	271.4609
Sample 4	10,357,620.5469	321.9609
Sample 5	976,691.5391	337.2188
Sample 6	74,317.2266	338.7422
Sample 7	124,840.8203	278.4219
Sample 8	8,590,618.125	285.2266
Sample 9	2,742,802.5703	279.125
Sample 10	2,761,672.8281	254.8906

### Differential attack analysis

Differential attack analysis^17^ is used to analyse the effectiveness of the encryption algorithm. It uses two cipher images. One ciphertext C1 is obtained from the original image, and another ciphertext C2 is obtained by changing one bit of the original image. Thus, the Number of Pixels Changing Rate (NPCR) and Unified Average Change Intensity (UACI) are calculated using the two-cipher text C1 and C2. The NPCR and UACI mathematical expressions are given in Eqs. (25) and (26).

25 $$NPCR = \frac{{\mathop \sum \nolimits_{i = 1}^{M} \mathop \sum \nolimits_{j = 1}^{N} D_{ij} }}{M \times N}$$

26 $$UACI = \frac{{\mathop \sum \nolimits_{i = 1}^{M} \mathop \sum \nolimits_{j = 1}^{N} \left| {C1\left( {i,j} \right) - C2\left( {i,j} \right)} \right|}}{M \times N}$$

where,

$$D_{ij} = \left\{ {\begin{array}{*{20}c} {0, C1\left( {i,j} \right) = C2\left( {i,j} \right)} \\ {1, C1\left( {i,j} \right) \ne C2\left( {i,j} \right)} \\ \end{array} } \right.$$

**Fig. 10:**
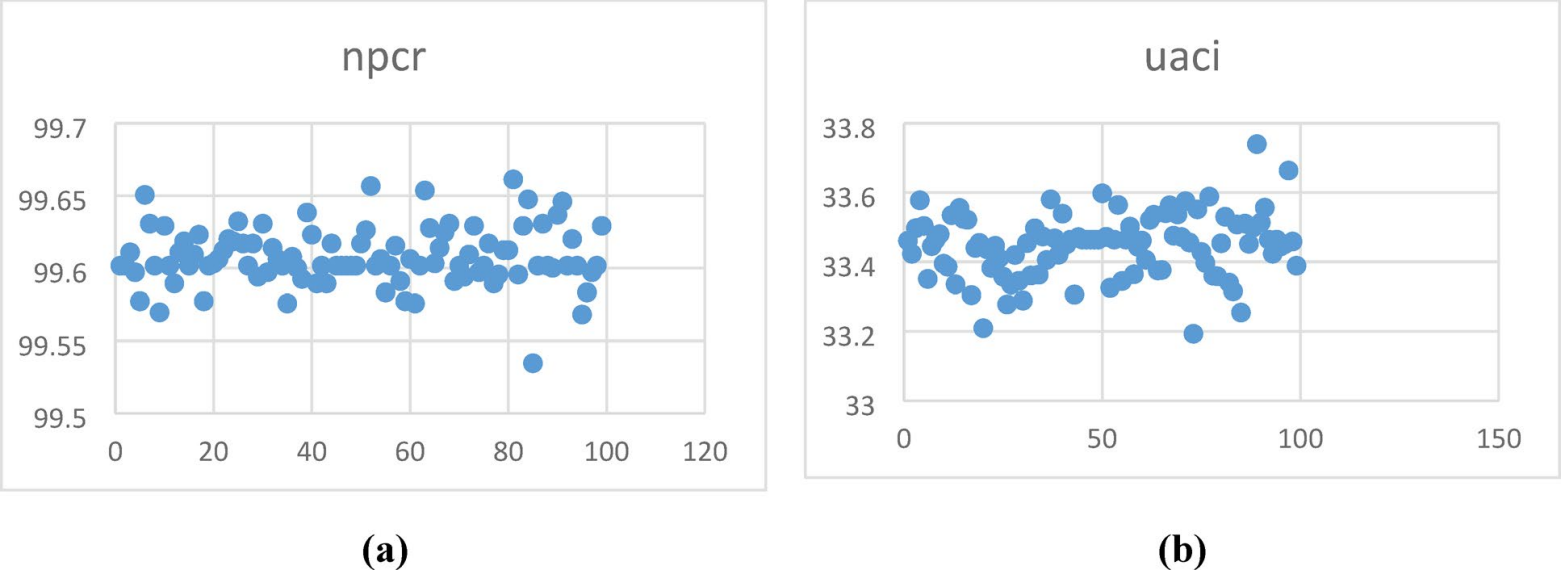
(**a**) NPCR, (**b**) UACI analysis of 100 samples.

M and N represent the size of the matrix. Figure 10 shows the NPCR and UACI values analysed for sample images, yielding an average NPCR value of 99.99% and a UACI value of 33.34%. The NPCR and UACI, compared with their critical values, are listed in Tables 6 and 7**.**

**Table 6:** NPCR analysis.

Dicom images	NPCR (%)	NPCR critical values		
		N*0.05 = 99.5693%	N*0.01 = 99.5527%	N*0.001 = 99.5341%
Sample 1	99.6017	Pass	Pass	Pass
Sample 2	99.6017	Pass	Pass	Pass
Sample 3	99.6109	Pass	Pass	Pass
Sample 4	99.5972	Pass	Pass	Pass
Sample 5	99.5773	Pass	Pass	Pass
Sample 6	99.6506	Pass	Pass	Pass
Sample 7	99.6307	Pass	Pass	Pass
Sample 8	99.6017	Pass	Pass	Pass
Sample 9	99.5697	Pass	Pass	Pass
Sample 10	99.6292	Pass	Pass	Pass

**Table 7:** UACI analysis.

Samples	UACI	UACI critical values		
		U*^-^_0.05_ = 33.2824% U*^+^_0.05_ = 33.6447%	U*^-^_0.01_ = 33.2255% U*^+^_0.01_ = 33.7016%	U*^-^_0.001_ = 33.1594% U*^+^_0.001_ = 33.7677%
Sample 1	33.4609	Pass	Pass	Pass
Sample 2	33.4233	Pass	Pass	Pass
Sample 3	33.4972	Pass	Pass	Pass
Sample 4	33.5778	Pass	Pass	Pass
Sample 5	33.5037	Pass	Pass	Pass
Sample 6	33.3507	Pass	Pass	Pass
Sample 7	33.4453	Pass	Pass	Pass
Sample 8	33.4641	Pass	Pass	Pass
Sample 9	33.4798	Pass	Pass	Pass
Sample 10	33.3945	Pass	Pass	Pass

### Brute force attack analysis

The encryption algorithm is validated for resisting brute force attacks in terms of key space and key sensitivity.

#### Keyspace analysis

A brute force attack^3^ is cracking/ stealing information by trying out all possible combinations^18^. The proposed encryption algorithm is said to be strong only if its keyspace is larger than 2^128^. Our proposed encryption algorithm uses 7 control parameters and 5 initial states. Also, it uses SHA-512 to generate a hash value for the initial seed parameters. So total, 12 parameters are used with 15-bit precision. So, the total key space used in the encryption algorithm is 10^12×15^ × 2^512^≈ 10^180^ × 2^512^, larger than 2^128^.

#### Key sensitivity analysis

**Fig. 11:**
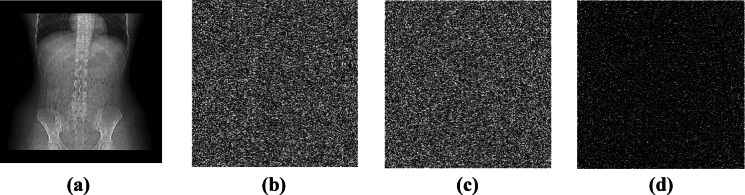
(**a**) Original image, (**b**) Encrypted image using original key, (**c**) Encrypted image using varied key, (**d**) Difference between b and c.

The sensitivity of the encryption algorithm is strategised through the key sensitivity analysis. A single bit change in the key, results in a different encrypted image. The proposed encryption algorithm’s key sensitivity performance is performed by making two different keys K1 and K2, where K1 is the key with initial values(unaltered) and K2 is the key by adding 0.0001 to its initial parameters(altered). This enumerates the sensitivity nature of the algorithm by producing different encrypted image. Figure 11 shows the sensitivity performance analysis of (a) original image, (b) encrypted image, (c) varied key encrypted image and (d) difference between the encrypted and the varied encrypted image. Therefore, it is observed from the scenario about the encryption algorithm is sensitive to its key.

### Error metric analysis

#### Mean square error (MSE)

The squared error between the encrypted and original images is calculated using the Mean Squared Error (MSE). The MSE is calculated using Eq. (27).

27 $$MSE = \frac{1}{M \times N}\mathop \sum \limits_{i = 0}^{M - 1} \mathop \sum \limits_{j = 0}^{N - 1} \left| {OI\left( {i,j} \right) - CI\left( {i,j} \right)} \right|^{2}$$

where M and N represents the size of the image. $OI\left( {i,j} \right)$ represents the original image and $CI\left( {i,j} \right)$ denotes the encrypted image. A sample of 10 images MSE and PSNR values are listed in Table 8

**Table 8:** MSE and PSNR analysis.

Dicom images	MSE	PSNR
Sample 1	17,187.6811	5.7786
Sample 2	17,891.6718	5.6043
Sample 3	14,632.1035	6.4777
Sample 4	19,411.3357	5.2502
Sample 5	12,966.0166	7.0027
Sample 6	8421.9576	8.8767
Sample 7	6737.8294	9.8456
Sample 8	21,836.6775	4.7389
Sample 9	14,676.4404	6.4646
Sample 10	14,631.0451	6.4781

#### Peak signal to noise ratio (PSNR)

The peak signal to noise ratio (PSNR) is calculated from the MSE in Section “Mean square error (MSE)”. The MSE value is applied to obtain a high PSNR value. The high PSNR value, close to 8, indicates the good quality of the image. The mathematical expression of PSNR is given in Eq. (28).

28 $$PSNR = 10\log_{10} \frac{{255^{2} }}{MSE}$$

**Fig. 12:**
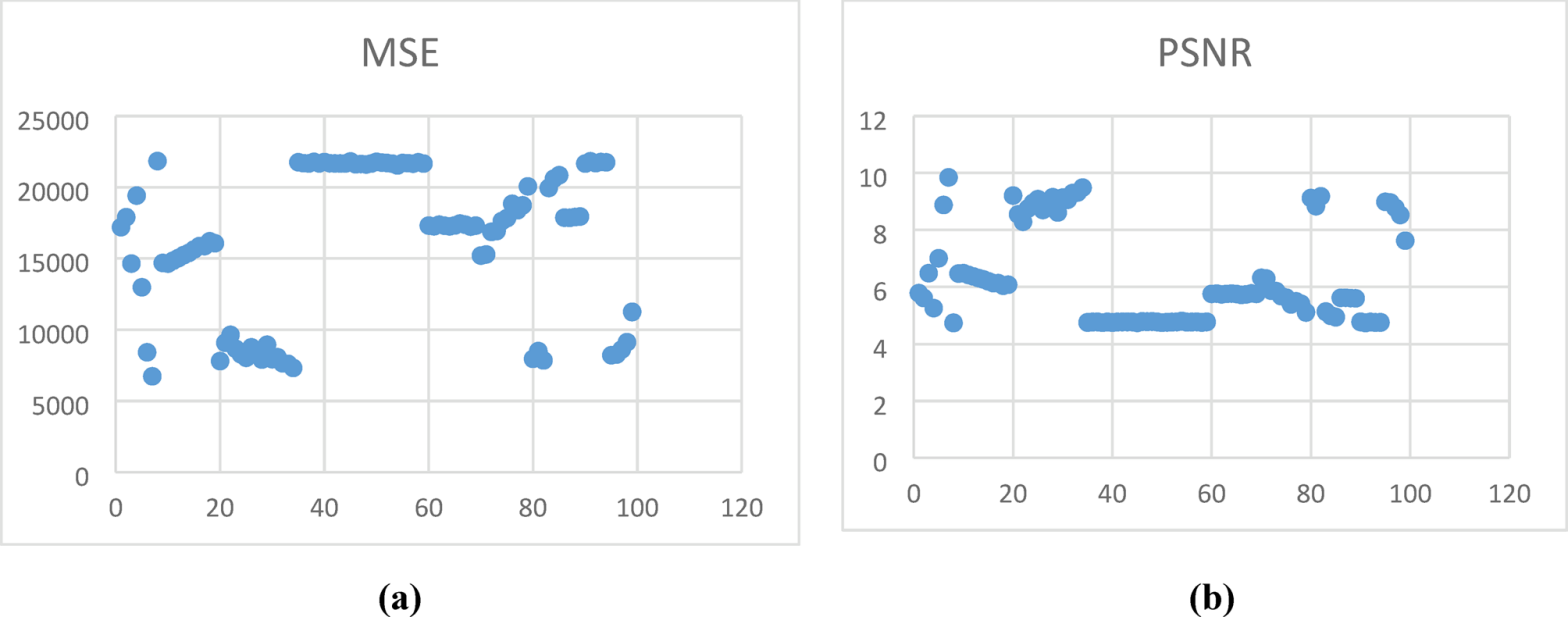
(**a**) MSE and (**b**) PSNR analysis of 100 samples.

The analysis has made for 100 samples and the respective MSE and PSNR values are plotted in Fig. 12

### Encryption quality analysis

The encryption algorithm quality^15^ is predominantin checking for security purposes. The quality can be evaluated through the original and the encrypted image histograms. It can be validated through metrics such as maximum deviation, uniform histogram deviation, and irregular deviation.

#### Maximum deviation

**Fig. 13:**
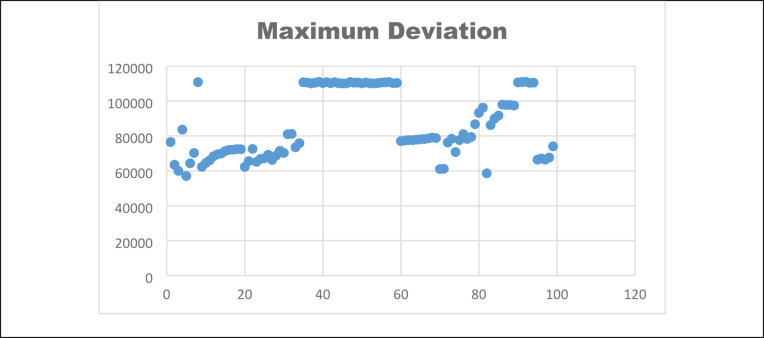
Maximum deviation.

The maximum deviation between the original and cipher image histograms is the deviation. It is calculated through the Eq. (29).

29 $$Max\_Dev = \frac{{K_{0} - K_{L - 1} }}{2} + \mathop \sum \limits_{i = 1}^{L - 2} K_{i}$$

where $K_{i}$ ith the difference between the original histogram and the cipher image histogram. Thus, the maximum deviation should be high to obtain higher security of the encryption algorithm. A 100 image samples are analysed and their points are plotted in Fig 13. Table 9 shows the maximum deviation values for 10 samples.

**Table 9:** Maximum deviation, deviation from ideality and irregular deviation values.

Sample images	MH	DI	ID
Sample 1	76,515.5	0.0571	68,504.97
Sample 2	63535S	0.0537	79,662.77
Sample 3	59,985	0.0521	64,038.59
Sample 4	83,660	0.057	107,574.5
Sample 5	57,048.5	0.0559	42,409.56
Sample 6	64,352	0.0576	19,540.83
Sample 7	70,285.5	0.0521	35,393.61
Sample 8	110,847	0.0529	127,994.3
Sample 9	62,336.5	0.0605	64,810.25
Sample 10	64,510	0.0496	42,442.55

**Fig. 14:**
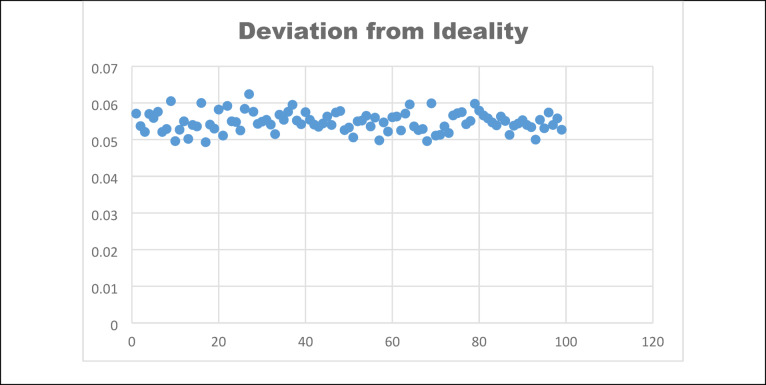
Deviation from ideality.

***Deviation from ideality ***It is said that the encrypted image histogram should be uniformly distributed for a good encryption algorithm. It is formulated using Eq. (30)

30 $$AH = \left\{ {\begin{array}{*{20}c} {\frac{M \times N}{{256}}, 0 \le L \le 255} \\ {0,elsewhere } \\ \end{array} } \right.$$

where AH is the solute histogram value and deviation from ideality is evaluated using the absolute and original histogram values. Hence, for a secured encryption algorithm, deviation from ideality should be less. Fig 14 shows that the analysis has been made for 100 samples to find the DI

31 $$DI = \frac{{\mathop \sum \nolimits_{i = 0}^{L} \left| {AH - OD} \right|}}{H \times N}$$

where AH is the absolute value histogram, and OD is the original image histogram, it is interpreted that the encryption algorithm’s DI values for 100 samples are the minimum. A sample 10 values is listed in Table 9

$$DI = \frac{{\mathop \sum \nolimits_{i = 0}^{L} \left| {AH - OD} \right|}}{H \times N}$$

**Fig. 15:**
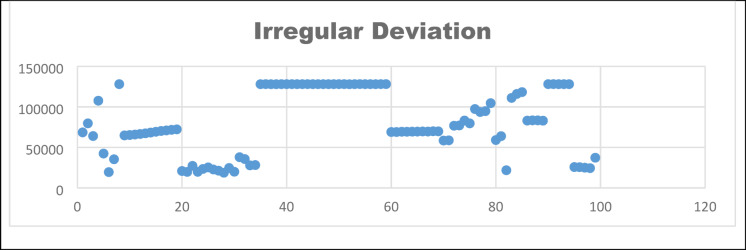
Irregular deviation.

*Irregular deviation *Irregular deviation helps to find the ability to use encryption. It is calculated through the eq (32).

32 $$ID = \mathop \sum \limits_{i = 0}^{N - 1} D$$

33 $$D = \left| {di - mean\left( H \right)} \right|$$

where D represents the difference of the original and encrypted image histogram and mean(H) denoted the average of all the histogram values. To evaluate the proposed encryption algorithm, the irregular deviation value should be high. Fig 15 shows the irregular deviation for 100 samples.

### Cropping and noise attack analysis

**Fig. 16:**
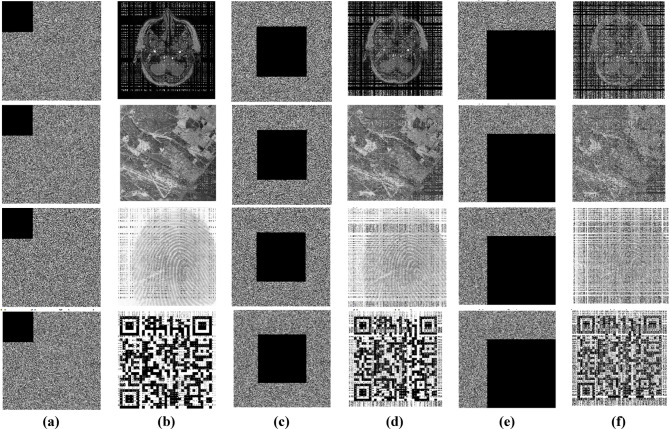
Cropping attack analysis (**a**)encrypted image cropped with 10%, (**b**) decrypted image of a, (**c**)encrypted image cropped with 25%, (**d**) decrypted image of c, (**e**)encrypted image cropped with 50%, (**f**) decrypted image of e.

To analyse the robustness of the encryption algorithm, a portion of the encrypted image is intentionally cropped or removed to check whether the cryptosystem can obtain information from the decrypted one, even after the cropping. To analyse the performance, the encrypted images are cropped at ratios of 10%, 25%, and 50%. The DICOM, satellite, fingerprint and QR code images are used in this analysis. Figure 16 shows the cropping attack analysis (a) encrypted image cropped with 10% (b) decrypted images of a, (c) encrypted image cropped with 25% (d) decrypted images of c, (e) encrypted image cropped with 50% (f) decrypted images of e.

**Fig. 17:**
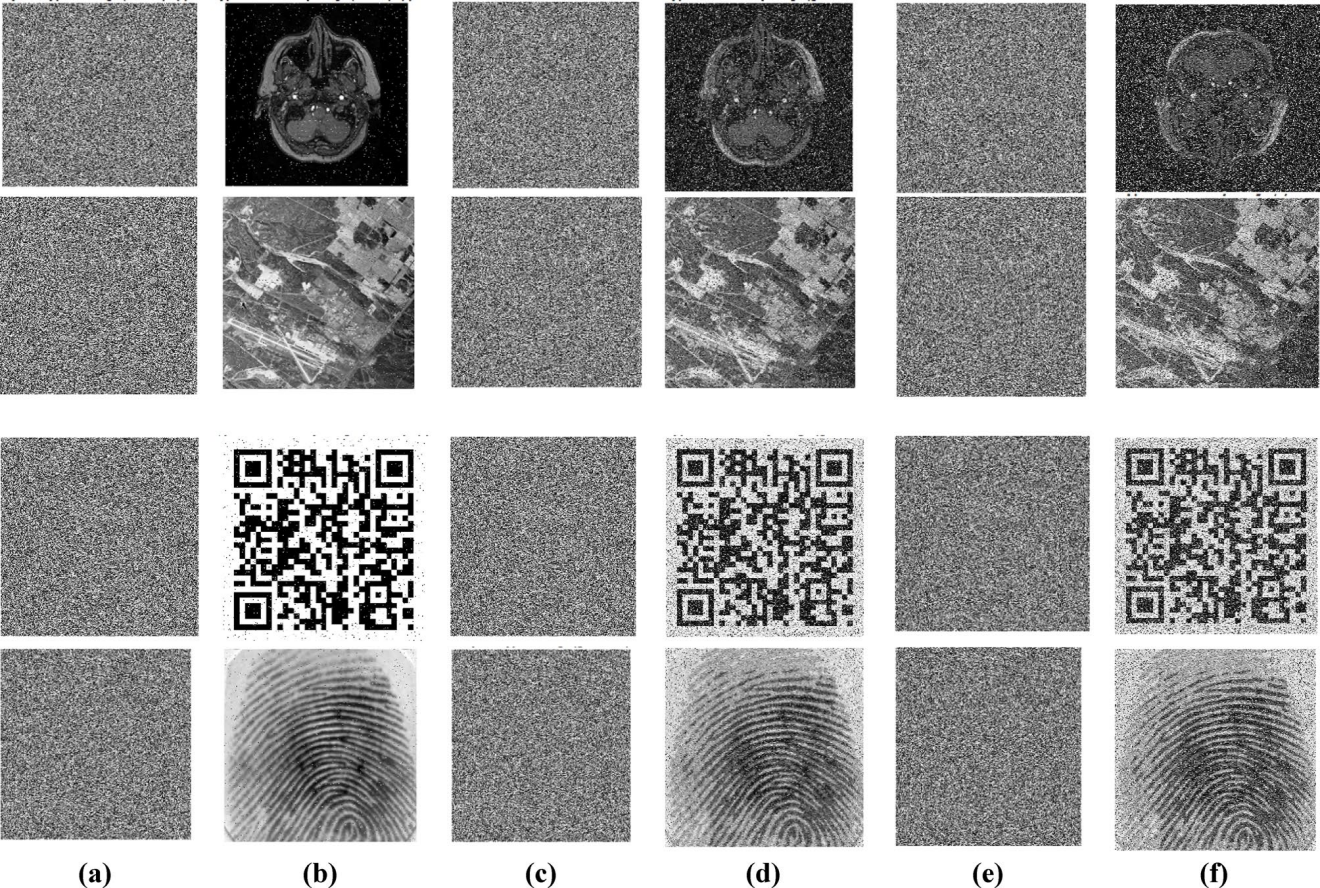
Noise attack analysis (**a**) encrypted image added with salt and pepper noise, (**b**) decrypted image of a, (**c**) encrypted image added with Gaussian noise, (**d**) decrypted image of c, (**e**) encrypted image added with speckle noise, (**f**) decrypted image of e.

Similarly, for noise attack analysis, various types of noise, such as salt and pepper, Gaussian, and speckle noises, are added to the encrypted images to analyse the behaviour of the cryptosystem. Salt, pepper, Gaussian, and speckle noises are added to the encrypted images to evaluate this process. Figure 17 shows the noise attack analysis: (a) salt and pepper noise added with the ratio 0.02 to the encrypted image, (b) decrypted image of a, (c) gaussian noise added with the variance 0.005 to the encrypted image, (d) decrypted image of c, (e) Speckle noise added with the variance of 0.04 to the encrypted image, (f) decrypted image of e.

### Complexity analysis

The complexity required for the initialisation is O(1). Similarly, for chaotic sequence generation, it requires O(1) time. The steps of initial diffusion, row shuffling, XOR-ing after row shuffle, column shuffling, XOR-ing after column shuffle, and the final step operation with the secondary image require O(MN). So, the total time complexity for the proposed encryption algorithm is O(MN). This is computationally efficient and scalable for practical images. Additionally, the average encryption time determined for the proposed work is 0.5324 s, and the decryption time is 0.4871 s. This states that our proposed encryption algorithm is computationally reasonable, hence it can be applied to real-time scenarios.

### Comparative analysis

As we have compared our proposed methodology with existing literature correlations, NPCR, UACI, entropy, and keyspace. Table 10 presents a summary of the comparative results. From the comparative analysis, it is clear that the proposed encryption is well-suited for all types of file images and has a larger key space, which resists brute force attacks. The NPCR and UACI also depict a strong sensitivity towards differential attacks.

**Table 10:** Comparative analysis.

Literature	HC	VC	DC	NPCR (%)	UACI (%)	Entropy	Keyspace
Niu et al.^10^	0.0018	0.0020	0.0001	99.6735	33.2765	7.9974	10^60^
Nan-Run et al.^19^	0.0040	0.0037	0.0030	99.6089	33.4658	7.9985	10^142^
Mingxu et al.^20^	0.003972	0.0002		99.6093	33.4641	7.9973	2^364^
Ying et al.^21^	0.0018	0.0020	0.0001	99.6735	33.2765	7.994	10^60^
WanQing et al.^22^	− 0.0093	− 0.0006	0.0004	99.62	33.48	7.9967	2^256^ × 10^16^
Sanjay et al.^23^	0.0022	0.0014	0.0018	99.98	33.44	7.9993	NA
Zeben et al.^24^	− 0.0344	0.0298	− 0.0599	99.61	33.62	7.9895	10^240^
Lizong Li et al.^25^	− 0.0013	0.0016	0.0015	NA	NA	7.9993	10^60^
Mingxu et al.^26^	0.0011	0.0012	0.0042	99.6140	33.4663	7.9993	10^75^
Meng-meng et al.^27^	0.0013	0.0010	0.0012	99.6078	33.5708	7.9972	10^110^
Xin-li xu et al.^10^	0.0006	0.00004	− 0.0010	99.6084	33.4620	7.9998	2^471^
Asha J et al.^28^	− 0.0023	− 0.0009	− 0.0008	99.6134	33.4919	7.9974	> 2^512^
Nidhi et al.^29^	− 0.0073	− 0.0005	− 0.0022	99.62	NA	7.9982	NA
Wassim et al.^30^	− 0.003928	− 0.002473	− 0.00664	99.6076	30.6095	NA	2^10524^
Minxiu et al.^31^	− 0.00017	− 0.00097	− 0.00017	NA	NA	7.9993	10^127^
Yong et al.^32^	− 0.0310	0.00929	0.01164	99.6085	33.4748	7.9971	NA
Mengjiao et al.^33^	− 0.0024	− 0.0165	− 0.00089	99.6175	33.5075	7.9915	NA
Yu-chi et al.^34^	− 0.041	− 0.0118	− 0.0049	99.6077	33.4643	7.99934	2.8639 × 10^250^
Shamsa et al.^35^	− 0.000626	− 0.000133	− 0.00188	99.63	33.46	7.9991	10^120^
Qianqian et al.^36^	0.000033	− 0.000623	− 0.00083	99.6119	33.348	7.99926	2^654^
Fan-Qi et al.^37^	0.000840	− 0.000464	− 0.02497	99.6012	33.412	7.9974	2^360^
Dhivya et al.^38^	0.00163	− 0.0012	0.00775	99.6081	33.4120	7.9958	2^856^
Lakshmi et al.^39^	0.0030	0.0025	− 0.0076	99.57	33.05	7.99457	10^112^
Janakiraman et al.^40^	0.0008	0.0046	0.0032	99.6613	33.5596	7.9976	2^7016^
Proposed	0.00025	0.0109	0.00955	99.6069	33.4284	7.99442	2^512^ × 10^180^

The robustness of the proposed encryption algorithm is validated by proving their efficiency by taking the performance metrics like NPCR, UACI and Entropy. The proposed algorithm attained the average NPCR value a 99.6069%, UACI as 33.4284% and entropy as 7.9944. While comparing with the other sample bench mark algorithms like Lakshmi et al.^34^ has its NPCR values as 99.57%, Fan-Qi et al.^37^ has 99.6012% and Ying et al.^21^ has 99.6735%. It shows that trivial alterations give strong sensitivity, where the values are close related to the ideal value. On taking the account of UACI, the existing algorithm from authors Niu et al.^10^ has its UACI value as 33.27%, Asha J et al.^28^ has 33.49%, and Qianqian et al.^36^ has 33.348% as its UACI value evidencing the proposed UACI is near to the ideal value and strong resistance against differential attacks. While discussing about the entropy value, the randomness and the uncertainty of the encrypted image is experimented in Mengjiao et al.^33^ has entropy value as 7.9915, Lizong Li et al.^25^ has 7.9993 and Yong et al.^32^ has its entropy value as 7.9971. Hence our proposed work entropy ensures strong confusion and diffusion, where it’s reaching its ideal value 8.

From the analysis, it is evident that the real-world applicability of the proposed cryptosystem is applicable to various applications, including healthcare, where real-time DICOM images are encrypted to ensure medical image security. In biometric security, we experimented with fingerprint images for secure authentication. We also validated the QR code images used in digital payment and IoT devices. Additionally, we explored geospatial data, specifically satellite images, which contain sensitive information. The proposed algorithm effectively demonstrates its robustness and effectiveness in various real-world applications through statistical attack analysis, as well as strong resistance against differential and brute force attacks.

## Conclusion and future work

In this paper, a multi-round confusion and diffusion algorithmic process has been performed by adopting a 5D modified hyperchaotic chaotic map equation. The merit of this research lies in its provision of a lightweight encryption module that offers security for real-time information. Thus, the encryption process deliberately uses chaotic sequences for pixel confusion and diffusion. The randomness of the chaotic sequence is evaluated by performing the NIST test. Various performance tests of the encryption algorithm are conducted. The simulation analysis was conducted on 100 images, including DICOM, satellite, fingerprint, and QR code. The resultant analysis demonstrates the robustness and security of image information across all file formats. Improvement would be concentrated on higher dimensions and real-world hardware implementation for future analysis.

Future research work could focus on streamlining hardware implementations to achieve higher throughput for real-time image encryption applications, notably in resource-constrained environments. The chaos-based encryption workflow can be extended to video data, addressing the unique challenges of temporal correlation between frames while maintaining computational efficiency. Subsequent work can be performed to investigate adaptive chaotic parameter selection mechanisms that could automatically optimise the system based on characteristics and security requirements. Implementing the proposed scheme on various hardware platforms, including FPGAs and custom ASICs, would validate its practical applicability in diverse scenarios. These advancements will further entrench chaotic cryptography as a viable alternative to conventional encryption methods, particularly for multimedia content where both security and performance are essential aspects.

## Declarations

### Data availability

The datasets generated during and/or analysed during the current study are available from the corresponding author upon reasonable request. The sources of the sample images are downloaded from https://sipi.usc.edu/database/database.php?volume=aerials, https://www.kaggle.com/datasets/samahsadiq/benign-and-malicious-qr-codes, https://www.dicomlibrary.com/, http://bias.csr.unibo.it/fvc2000/download.asp.

## References

[1] Corporation, I. Cost of a data breach report 2024. *IBM Secur.* 1–73 (2021).

[2] Liu, M., Ning, C. & Zhu, C. A secure image encryption scheme based on a new hyperchaotic system and 2D compressed sensing. *Entropy* **26**, 1–20 (2024). 10.3390/e26070603 PMC11276310 39056965

[3] Mansouri, A. et al. A secure medical image encryption algorithm for IoMT using a Quadratic-Sine chaotic map and pseudo-parallel confusion-diffusion mechanism. *Expert Syst. Appl.* **270**, 126521 (2025).

[4] Jain, S., Samal, A., Sharma, A., Khurana, M. & Sharma, B. Advanced image security through chaotic system-based encryption and fisher-yates matrix shuffling. *Optik* **327**, 172304 (2025).

[5] Benyahia, K., Khobzaoui, A. & Benbakreti, S. Hybrid image encryption: leveraging DNA sequencing and Lorenz chaotic dynamics for enhanced security. *Clust. Comput.* **28**, 218 (2025).

[6] Wang, S., Peng, Q. & Du, B. Chaotic color image encryption based on 4D chaotic maps and DNA sequence. *Opt. Laser Technol.* **148**, 107753 (2022).

[7] Singh, D., Kaur, H., Verma, C., Kumar, N. & Illés, Z. A novel 3-D image encryption algorithm based on SHA-256 and chaos theory. *Alex. Eng. J.* **122**, 564–577 (2025).

[8] Cheng, X. F., Zhu, H., Liu, L., Mao, K. & Liu, J. Dynamic analysis of a novel hyperchaotic system based on STM32 and application in image encryption. *Sci. Rep.* **14**, 1–21 (2024). 39227661 10.1038/s41598-024-71338-x PMC11372127

[9] Alawida, M. A novel chaos-based permutation for image encryption. *J. King Saud. Univ. Comput. Inf. Sci.* **35**, 101595 (2023).

[10] Xu, X. L., Song, X. G., Liu, S. H., Zhou, N. R. & Wang, M. M. New 2D hyperchaotic cubic-tent map and improved 3D Hilbert diffusion for image encryption. *Appl. Intell.* **55**, 590 (2025).

[11] Gao, S. et al. A 3D model encryption scheme based on a cascaded chaotic system. *Signal Process.* **202**, 108745 (2023).

[12] Lai, Q., Zhu, C., Qin, M. & Wan, Z. Complex dynamics and encryption application of a 3D dual-memristor oscillatory hyperchaotic map. *Math. Comput. Simul.* **236**, 270–283 (2025).

[13] Subathra, S. & Thanikaiselvan, V. Enhanced security for medical images using a new 5D hyper chaotic map and deep learning based segmentation. *Sci. Rep.* **15**, 22628. 10.1038/s41598-025-04906-4 (2025). 40593969 10.1038/s41598-025-04906-4 PMC12215922

[14] Arthi, G., Thanikaiselvan, V. & Amirtharajan, R. 4D Hyperchaotic map and DNA encoding combined image encryption for secure communication. *Multimed. Tools Appl.* **81**, 15859–15878 (2022).

[15] Arif, J. et al. A novel chaotic permutation-substitution image encryption scheme based on logistic map and random substitution. *IEEE Access* **10**, 12966–12982 (2022).

[16] Tang, S., Xu, X., Jiang, Z., Meng, D. & Sun, K. An image encryption scheme without additional key transmission based on an N-dimensional closed-loop coupled triangular wave model. *Chaos Solitons Fractals* **185**, 115039 (2024).

[17] Lai, Q., Hu, G., Erkan, U. & Toktas, A. A novel pixel-split image encryption scheme based on 2D Salomon map. *Expert Syst. Appl.* **213**, 118845 (2023).

[18] Al-Dayel, I., Nadeem, M. F., Khan, M. A. & Abraha, B. S. An image encryption scheme using 4-D chaotic system and cellular automaton. *Sci. Rep.* **15**, 1–22 (2025). 40461753 10.1038/s41598-025-95511-y PMC12134365

[19] Zhou, N. R., Hu, L. L., Huang, Z. W., Wang, M. M. & Luo, G. S. Novel multiple color images encryption and decryption scheme based on a bit-level extension algorithm. *Expert Syst. Appl.* **238**, 122052 (2024).

[20] Wang, M. et al. A new 2D-HELS hyperchaotic map and its application on image encryption using RNA operation and dynamic confusion. *Chaos Solitons Fractals* **183**, 114959 (2024).

[21] Niu, Y., Zhou, H. & Zhang, X. Image encryption scheme based on improved four-dimensional chaotic system and evolutionary operators. *Sci. Rep.* **14**, 1–22 (2024). 38528145 10.1038/s41598-024-57756-x PMC10963803

[22] Wu, W. Q. & Kong, L. S. Image encryption algorithm based on a new 2D polynomial chaotic map and dynamic S-box. *Signal Image Video Process.* **18**, 3213–3228 (2024).

[23] Kumar, S. & Sharma, D. Image scrambling encryption using chaotic map and genetic algorithm: A hybrid approach for enhanced security. *Nonlinear Dyn.* **112**, 12537–12564 (2024).

[24] Zhuang, Z., Zhuang, Z. & Wang, T. Medical image encryption algorithm based on a new five-dimensional multi-band multi-wing chaotic system and QR decomposition. *Sci. Rep.* **14**, 1–17 (2024). 38172586 10.1038/s41598-023-50661-9 PMC10764812

[25] Li, L. Image encryption algorithm based on hyperchaos and DNA coding. *IET Image Process.* **18**, 627–649 (2024).

[26] Wang, M. et al. A new 2D cross hyperchaotic sine-modulation-logistic map and its application in bit-level image encryption. *Expert Syst. Appl.* **261**, 125328 (2025).

[27] Wang, M. M., Song, X. G., Liu, S. H., Zhao, X. Q. & Zhou, N. R. A novel 2D Log-Logistic–Sine chaotic map for image encryption. *Nonlinear Dyn.* **5**, 2867–2896 (2024).

[28] Vithayathil, A. J. & Sreekumar, A. Novel encryption method for color images based on cross-channel substitution and permutation using an improved two-dimensional Henon map (2D IHM). *Nonlinear Dyn.*10.1007/s11071-024-10736-2 (2024).

[29] Khurana, N. & Dua, M. A novel one-dimensional Cosine within Sine chaotic map and novel permutation–diffusion based medical image encryption. *Nonlinear Dyn.* **113**, 4839–4859 (2024).

[30] Alexan, W. et al. A new multiple image encryption algorithm using hyperchaotic systems, SVD, and modified RC5. *Sci. Rep.* **15**, 1–33 (2025). 40118872 10.1038/s41598-025-92065-x PMC11928741

[31] Yan, M., Liu, M. & Li, C. DNA color image encryption based on conservative chaotic system. *Sci. Rep.* **15**, 1–19 (2025). 40102601 10.1038/s41598-025-93649-3 PMC11920197

[32] Chen, Y., Xie, S. & Zhang, J. A hybrid domain image encryption algorithm based on improved Henon map. *Entropy* **24**, 287 (2022). 35205581 10.3390/e24020287 PMC8870770

[33] Wang, M., Yi, Z. & Li, Z. A memristive Ikeda map and its application in image encryption. *Chaos Solitons Fractals* **190**, 115740 (2025).

[34] Lan, Y. C. & Wang, C. M. A novel multi-image encryption scheme using generalized rectangular transform and advanced 5-D Hyperchaotic map. *IEEE Access* **13**, 43316–43337 (2025).

[35] Kanwal, S. et al. An efficient image encryption algorithm using 3D-cyclic chebyshev map and elliptic curve. *Sci. Rep.* **14**, 1–15 (2024). 39609493 10.1038/s41598-024-77955-w PMC11605050

[36] Shi, Q., Qu, S., An, X., Wei, Z. & Zhang, C. Three-dimensional m-HR neuron model and its application in medical image encryption. *Chaos, Solitons Fractals* **189**, 115701 (2024).

[37] Meng, F. Q. & Wu, G. A color image encryption and decryption scheme based on extended DNA coding and fractional-order 5D hyper-chaotic system. *Expert Syst. Appl.* **254**, 124413 (2024).

[38] Ravichandran, D. et al. An efficient medical data encryption scheme using selective shuffling and inter-intra pixel diffusion IoT-enabled secure E-healthcare framework. *Sci. Rep.* **15**, 4143 (2025). 39900990 10.1038/s41598-025-85539-5 PMC11790928

[39] Lakshmi, C. et al. Reconfigurable security solution based on hopfield neural network for e-healthcare applications. *Sci. Rep.* **15**, 5628 (2025). 39955320 10.1038/s41598-025-88561-9 PMC11830060

[40] Janakiraman, S. et al. Integrity verified lightweight ciphering for secure medical image sharing between embedded SoCs. *Sci. Rep.* **15**, 1–21 (2025). 40032946 10.1038/s41598-025-91431-z PMC11876590

